# Fungal morphotype detection and quantification in microscopic images with TU_MyCo-vision: a user-friendly deep learning object detection tool

**DOI:** 10.1186/s40694-026-00215-1

**Published:** 2026-08-11

**Authors:** Kartik J. Deopujari, Matthias Schmal, Caroline Danner, Zainab Abdul Qayyum, Jordy T. Zwerus, Julian Kopp, Mihail Besleaga, Roghayeh Shirvani, Astrid R. Mach-Aigner, Robert L. Mach, Christian Zimmermann

**Affiliations:** Institute of Chemical, Environmental and Bioscience Engineering, TU Wien, Gumpendorfer Strasse 1a, Wien, 1060 Austria

**Keywords:** Fungal morphology, Deep learning, YOLO object detection, Brightfield microscopy, *Aureobasidium pullulans*, Automated image analysis

## Abstract

Morphological switching in response to environmental stimuli is a well-known phenomenon in fungi, leading to diverse morphotypes. Microscopic observation remains a widely used approach to study these phenotypes, but variation in sample preparation and operator skill can limit the scale of sample processing or introduce operator bias. Although several image-based cell detection tools have been developed, most are tailored to specific applications or limited to a particular taxon. To address the need for a tool applicable to the polymorphic, yeast-like fungus *Aureobasidium pullulans*, and with potential applicability to other taxa, we developed TU_MyCo-Vision, an Ultralytics YOLO (You Only Look Once) based object detection tool for identifying 13 fungal morphotypes in bright-field microscopic images. Identification of 13 fungal morphotypes, including variation of vacuolated single cells, cells with granular cytoplasmic appearance, and diverse hyphal forms, is achieved by integrating a YOLOv11m-based object detector trained on a custom dataset of 1,504 annotated images and a standalone graphical user interface that enables downstream data analysis and visualization of results. The best-performing model (Zulu_s3) achieved a mean precision of 73.4%, a recall of 66.5%, a mean average precision at 50% IoU (mAP@50) of 73.5%, and a mean average precision at varying IoU thresholds between 50 and 90% IoU (mAP@50–95) of 54.5% across all 13 classes. The single-group analysis pipeline was validated on a 90-image test set, generating six quantitative summaries that capture the distribution and co-occurrence of fungal morphotypes, including absolute counts, relative and mean relative abundance plots, stacked bar plots and clustered heatmaps. Multi-group evaluation on previously unseen datasets comprising *Candida albicans*, *Komagataella phaffi*, and *Aspergillus niger* spores demonstrated that these morphotype profiles can be compared across biologically distinct genera, highlighting the tool’s potential applicability for studying fungal morphological diversity.

**Supplementary Information:**

The online version contains supplementary material available at 10.1186/s40694-026-00215-1.

## Background

### Morphological plasticity in fungi

 The kingdom of fungi is well known for its diversity, especially in modes of nutrition, habitats, and morphology. An interesting phenomenon in some fungi is the ability to exhibit different morphologies throughout their life cycle. These morphological changes may occur in response to environmental stimuli or contribute to pathogenicity [1]. A good example is morphological switching in *Candida albicans*, where the hyphal and pseudo-hyphal morphologies are generally considered pathogenic, whereas the yeast form is regarded as commensal [1, 2]. A similar tendency to switch morphology is also observed in soil-borne pathogenic fungi *Coccidioides immitis* and *Coccidioides posadasii*, in which spherules form through the progressive enlargement of arthroconidia that enter the body via inhalation [3]. This morphological switch is also an important factor in treatment decisions between *Aspergillus* and *Mucorales* infections, based on the presence of septate versus non-septate hyphae, as assessed by micromorphology and microscopy [4].

Beyond its clinical dimension, fungal polymorphism is equally significant in a biotechnological context. *Aureobasidium pullulans*, a polyextremotolerant black yeast-like fungus, is a good example of morphological plasticity in an industrially relevant organism. *A. pullulans* is well known for producing a polymer pullulan, along with other metabolites such as heavy oils and polymalic acid [5]. It is known to survive across a wide spectrum of extreme environments, including hypersaline, highly acidic or basic, cold and oligotrophic conditions. However, polymorphism is not limited to A. pullulans but is observable throughout the genus Aureobasidium. This environmental response leads to the development of various morphotypes, which include yeast-like cells, blastoconidia, chlamydospores, hyphae, pseudohyphae, swollen cells, and septate cells [6, 7]. Li et al. described several morphological forms in *A. pullulans* NG, including yeast-like blastospores (YLB), yeast-like cells (YL), swollen cells (SC), septate swollen cells (SSC), chlamydospore-like cells (CH), and meristematic structures (MS) [8]. Among these, the SC form plays a key role, acting as a relatively stable intermediate that arises from YL cells. This form is notable for producing key metabolites, such as unpigmented pullulan, and subsequently differentiating into melanin-rich forms, such as CH, HY, and MS, as the organism progresses into later growth stages. A practical application of this was demonstrated by Zeng et al., who showed the role of citric acid in controlling cellular differentiation in A. pullulans NG, leading to efficient pullulan biosynthesis due to cell growth in swollen cell form [9].

### Microscopic assessment of fungal morphology and computer vision applications

Microscopic observation of fungal morphology remains one of the most commonly used methods for phenotype assessment [10]. In clinical settings, staining techniques such as potassium hydroxide (KOH), calcofluor white, and Gram stain allow rapid detection of fungal elements, including yeast cells, budding cells, hyphae, and pseudohyphae, in specimens such as bronchoalveolar lavage, skin scrapings, and cerebrospinal fluid [4]. The distinction between septate and non-septate hyphal structures can guide antifungal therapy, and the presence of fungal elements in sterile body sites constitutes independent proof of invasive infection. However, accurate interpretation depends heavily on operator expertise, sample preparation quality, and awareness of artefacts that may simulate fungal morphology [4]. In research settings, these limitations are compounded: manual assessment of morphotype distributions across hundreds or thousands of microscopic images is inherently labour-intensive, subjective, and prone to inter-observer variability.

The convergence of computer vision, deep learning, and increased computational resources has driven the development of automated tools for fungal image analysis across applications ranging from crop disease surveillance and ecological monitoring to clinical microbiology and fundamental research. In plant pathology, machine learning approaches have been applied to classify spores of tomato disease-causing fungi using textural, colour, and shape descriptors [11], to identify *Lasiodiplodia* grapevine species using feature selection with a linear SVM [12], to detect *Fusarium* scab spores in wheat using segmentation-based deep learning [13], to count grey mould spores from cucumber plants using a multi-head self-attention YOLO architecture [14], and to classify spores of three *Trichoderma* species using random forest classifiers [15]. At broader taxonomic scales, CNN architectures have classified microscopic images spanning 89 fungal genera [16] and automated the identification of soil fungi from large brightfield image datasets acquired by fully automated robotic microscopy [17, 18]. In clinical microbiology, a YOLOv4 model trained on KOH images detected dermatophyte hyphae at sensitivities up to 99.0% [19], while classification networks identified *Candida* species from single-cell brightfield wet mount images [20], a cascaded YOLOv5 approach achieved slide-level diagnosis of vulvovaginal candidiasis matching or exceeding experienced human readers across multi-hospital datasets [21], and smartphone-acquired microscopic images of vaginal discharge were classified using ResNet18 in resource-limited settings [22]. For filamentous fungal keratitis, parallel binary classifiers distinguished *Fusarium* and *Aspergillus* from other filamentous genera in confocal microscopy images without requiring hypha-level annotations [23], a meta-learning framework outperformed multiple standard CNN architectures for detecting superficial fungal infections from dermatological images [24], and YOLOX combined with MobileNet V2 was applied to detect spores, hyphae, and mycelium in fluorescence microscopic images [25].

A good morphologically granular system is Candescence, a fully convolutional object detector trained on approximately 80,000 individually annotated *Candida albicans* cells, classifying nine distinct morphotype; yeast white, budding yeast, opaque, budding opaque, gray like, budding gray like, shmoo, pseudohypha, and hypha, using a curriculum-based learning strategy and achieving an adjusted F1 score of 83.2% [26]. Beyond medical mycology, automated detection has been applied to mycelial clamp connections and hyphal autolysis in food-technology contexts, with a web-based interface provided for user accessibility [27], and to field-microscopic detection of cucumber downy mildew spores under challenging real-world imaging conditions, achieving an AP of 0.956 with a lightweight model of only 2.8 M parameters [28].

### Limitations of existing systems and TU_MyCo-Vision

Collectively, these studies demonstrate that computer vision and deep learning can support fungal microscopy across settings ranging from plant pathology and soil ecology to clinical diagnostics. Yet most existing systems are optimized for specific defined tasks like binary detection of superficial hyphae in KOH preparations, slide-level diagnosis of vulvovaginal candidiasis, or classification of nine *C. albicans* morphologies in DIC images, and are typically constrained to a single species, a limited morphological repertoire, or a specific imaging modality. Tools that perform morphotype-resolved detection either depend on DIC or fluorescence microscopy and focus on a single organism, as in Candescence for *C. albicans*, or they collapse morphological diversity into a single “fungus” class under brightfield conditions and offer no integrated pathway from detection to quantitative analysis accessible to non-programmers.

This gap is particularly acute for *Aureobasidium* species, where quantitative morphotype, distributions are tightly coupled to cell differentiation trajectories, pullulan and polymalic acid biosynthesis, etc., and responses to environmental stimuli such as pH, carbon source, and osmotic stress.

To address this, we developed TU_MyCo-Vision, an Ultralytics YOLOv11m-based object detection model trained to recognize thirteen morphotypes in *Aureobasidium* spp. directly from standard brightfield microscopy images. The training corpus comprised 1,504 curated images, primarily of *A. pullulans*, supplemented with *A. melanogenum* and *Trichoderma reesei* to broaden morphological and taxonomic coverage; annotations were generated through a hybrid strategy combining expert manual labelling with model-assisted auto-labelling. Model performance was assessed via stratified 5-fold cross-validation, and generalization was probed on brightfield images of *Komagataella phaffii*, *Aspergillus niger*, and *Candida albicans*, genera absent from the training set.

To maximise accessibility of the tool, TU_MyCo-Vision is deployed within a cross-platform graphical user interface that integrates morphotype-level detection with an embedded statistical analysis pipeline, enabling morphotype quantification, single- and multi-group comparisons, class-specific grouping, and interactive visualisation directly from brightfield image data without requiring programming skills.

## Methods

### Fungal strains and culture conditions

All *Aureobasidium pullulans* strains used in this study (EXF-150, EXF-3374, EXF-3519, EXF-3750, EXF-4010, EXF-5628, EXF-6298, EXF-6519, EXF-8127, EXF-8128, EXF-10632, and NBB 7.2.1) [29, 30] were cultivated in *A. flavus* and *A. parasiticus* medium (AFP), *Archimycetes* medium (ARCH), Brain Heart Infusion medium (BHI), boiled rice medium, potato dextrose medium (PD), yeast peptone dextrose medium (YPD), M17 medium, malt extract medium (MEX), Muller Hinton broth (MH), and minimal medium. Refer to Table S1 for detailed medium composition. All media compositions were adapted from Westerdijk Laboratory 762 Manual Series No. 1 Fungal Biodiversity (2019) [31]. 100 ml Flasks containing 25 ml of the respective cultivation medium and strain were incubated for different time periods at incubation temperatures ranging from 14 °C to 34 °C and at different shaking speeds ranging from 200 to 220 rpm.

*Aureobasidium melanogenum* ATCC 42,023 was cultivated on MEX plates at 22 °C for 5 days. Yeast-like growth was scraped off with a sterile inoculation loop and resuspended in 0.8% NaCl. 25 ml of fresh medium was inoculated with the suspension, starting with an OD_600_ of 0.05, and incubated at 24 °C at 200 rpm for approximately 24–168 h.

*Trichoderma reesei* strains (NRRL 15709) [32] and Rut-C30 [33] were cultivated on PD agar plates at 30 °C until sporulation. The conidia were harvested and resuspended in 0.8%(w/v) NaCl, 0.05% (v/v)Tween-80. 6-well culture plates, with each well containing 5 ml of growth medium, were inoculated to a density of 10^9^ conidia/L. The cultures were incubated at 30 °C for 20 h under static conditions. Three types of media were used for cultivation: Mandels–Andreotti (MA) medium supplemented with either 1% (w/v) lactose or 1% (w/v) glucose as the sole carbon source, and MEX medium.

*Komagataella phaffii* GS115 was cultivated in a Minifors 2 bioreactor (maximum working volume 2 L; Infors HT, Basel, Switzerland). The pre-culture medium (100 mL L⁻¹ of sterilized 0.1 M potassium phosphate buffer, pH 6.0, containing 13.4 g L⁻¹ yeast nitrogen base without amino acids and with ammonium sulfate, 5 g L⁻¹ (NH₄)₂SO₄, 400 mg L⁻¹ biotin, 20 g L⁻¹ glycerol, and 100 µg mL⁻¹ Zeocin) was inoculated with a fresh cryo stock and incubated at 30 °C, 230 rpm for 24 h. The resulting pre-culture was used to inoculate the batch medium at 10% (v/v). Process control and feeding were managed using EVE software (Infors HT, Bottmingen, Switzerland), and the batch medium composition followed Spadiut et al. [34] The pH was monitored with an EasyFerm Plus sensor (Hamilton, Reno, NV, USA) and maintained at 5.0 by automatic addition of 12.5% NH₄OH, with 10% H₃PO₄ added manually if required. The temperature was kept at 30 °C, and aeration with a mixture of pressurised air and pure oxygen at 2 vvm ensured that dissolved oxygen (dO₂), measured using a Visiferm DO electrode (Hamilton, Reno, NV, USA), remained above 30%. After depletion of the carbon source in the batch phase, chemostat operation was initiated, with reactor volume held constant via an immersion tube connected to a bleed pump. During chemostat cultivation, samples were collected at alternating time points chosen based on morphological changes, which depend strongly on cultivation time and dilution rate, as detailed in [35], and microscopic analysis was used to monitor pseudohyphal growth.

*K. phaffii* strains CBS7435, ∆DAS1, ∆DAS2, ∆AOX1 were cultivated in M2 citrate-buffered media [36] supplemented with 20 mg/L thiamine pyrophosphate (TPP). A single yeast colony from a YPD (1% yeast extract, 2% peptone, 2% glucose, and 1.5% bacteriological agar) agar plate was inoculated into a 250 mL shake flask containing 50 mL of YPD broth, and the preculture was grown at 28 °C and 200 rpm. The samples for microscopy were taken from cultures grown in M2 citrate buffered media with 1% (v/v) methanol as the carbon source. The dataset from this cultivation will be referred to as *Komagataella phaffii* strain set_A in this article.

For *Aspergillus niger* conidia, an industrial strain was revived from a -80 °C glycerol stock and spotted onto minimal medium plates, which were then incubated at 30 °C for 7 days to ensure sufficient conidiation. Conidia were harvested by adding 10 mL 0.1% (v/v) Tween-20 onto the plate surface and gently scraping with a sterile cotton swab; the suspension was filtered through Miracloth to remove mycelial and agar debris, centrifuged at 3000×g, 4 °C, 15 min, resuspended in 10 mL 0.1% Tween-20, and centrifuged again before final resuspension in up to 10 mL 0.1% Tween-20. These conditions were chosen to obtain highly dispersed conidial suspensions with minimal hyphal fragments, allowing a clean assessment of spore morphotypes as an additional test case for the detector’s generalisability.

### Microscopic image acquisition

Brightfield images of *A. pullulans*,* T. reesei*,* K. phaffii strain set A*,* and A. niger* were acquired on a Nikon ECLIPSE E200 light microscope equipped with a BRESSER Mikrookular CMOS Full HD eyepiece camera (Art. No. 5913650) using brightfield optics. For *T. reesei*, images were recorded at total magnification of 100× and 200×, while *K. phaffii* strain set A was imaged at 100×, 200×, and 400× total magnification to ensure sufficient spatial resolution for small yeast cells. *A. niger* conidia were diluted in 0.1% (v/v) Tween-20 and observed in an improved Neubauer counting chamber at 200× total magnification to obtain well-dispersed spore fields suitable for object detection. Image acquisition and export for this setup were performed using Bresser CamLab Lite at a resolution of 1920 × 1080 pixels (width × height).

Microscopic analysis of *K. phaffii* GS115 samples from bioreactor cultivations was carried out on an Olympus CKX41 inverted microscope (Olympus Life Science, Tokyo, Japan) equipped with an IX2-SLP phase contrast slider and a Canon EOS 250D camera (Canon, Tokyo, Japan), allowing visualisation of yeast-like and pseudohyphal morphologies in unstained preparations.

*A. melanogenum* cultures were imaged using a Leica DMi8 microscope at 100×, 400×, and 630× total magnification, and images were exported at 2560 × 1920 pixels (width × height) using Leica Application Suite.

### Dataset generation

**Fig. 1:**
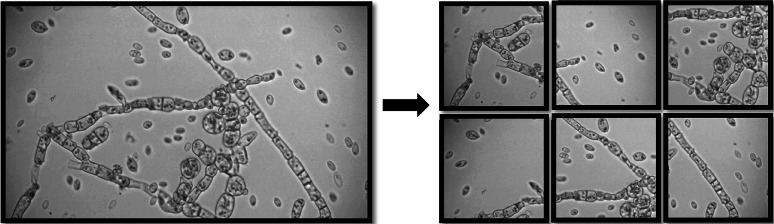
Representative full-size (1920 × 1080 pixels) microscopic image of *A.pullulans* (left panel) cropped into six unique 640 × 640 pixel crops (right panel) as part of the dataset expansion strategy

Exported 1920 × 1080 pixels images were cropped into 640 × 640 pixels tiles (Fig. 1), using a custom Python script “cropper.py”. In total, 15,437 cropped images of 640 × 640 px were generated and used as an image bank for annotation and dataset assembly.

Iteratively, three dataset versions were created: X-ray, Yankee and Zulu. To make efficient use of our datasets, we implemented 5-fold stratified cross-validation for each dataset family. A custom Python script (kfold_splitter.py), adapted from an Ultralytics cross-validation utility [37], was used to partition each family (X-ray, Yankee, Zulu) into five folds. Stratification was performed based on object count per class. An independent global test dataset of 166 images was created from the primary image bank. Images not part of any of the three datasets were included in the test dataset.

All 640 × 640 px tiles were annotated manually using LabelMe v5.4.1 by a trained annotator with experience in fungal microscopy [38].

Annotations were performed by drawing tight rectangular bounding boxes around the cells. The class assignment was based on the 13 morphological classes, determined from microscopic observations. To monitor class distributions during dataset construction and to support targeted class-balancing (e.g. enrichment of rare filamentous or special classes), a custom Python script “annotation handler.py” was used to extract per-image class counts from the LabelMe annotation files. The Python library Labelme2YOLO (v0.1.7) and Labelme2YOLO (v0.2.5) were used to convert annotations in “.JSON” format to “YOLO” annotation format. The dataset was automatically split into an 80:20 Train: Validation split during conversion [39]. For the Zulu dataset (final hybrid dataset), auto-labelling was performed on images from the primary image bank that were not included in any previous dataset, using a Python script (datafeed_prediction.py) to run the trained model (Yankee) prediction function and generate bounding-box overlays along with annotation text files in YOLO format. Post-prediction, all 14,200 candidate images were manually reviewed and added after meeting QC criteria [37]. 

### Image quality control and annotation scheme

All 640 × 640 images were manually inspected by the annotator and were annotated only if they fulfilled the following conditions.

1. Sharp enough focus to resolve cellular structures as defined in Results 1, “Morphological assessment and Class definitions”.
2. Illumination, exposure, and contrast had to allow clear discrimination of intracellular structures from the background.
3. Absence or minimal presence of non-biological artefacts like air bubbles, scratches on glass surfaces, and dust.
4. Images dominated by cells cut off at the image borders (“edge-only growth”), dense unresolved clumps (except when required to represent class C6, cell aggregates/clumps), or images without cells were removed from the dataset.

During annotation, bounding boxes were adjusted to tightly enclose individual cells or continuous filaments. For extensive hyphal networks spanning large image regions, we applied two complementary strategies to increase the diversity of training examples and better capture object scale: (i) when filaments occupied almost the entire field of view, a single large bounding box was drawn around the continuous structure, and (ii) for discontinuous but spatially extended hyphal clusters, multiple smaller bounding boxes were placed around subregions of the same filamentous structure. Clearly visible single cells within the large bounding boxes were labelled into their respective classes. Care was taken to avoid intersections within boxes, especially in single-cell boxes.

## Training TU_MyCo-vision with custom dataset

### Initial training and hyperparameter exploration

A pre-trained YOLOv8x model from Ultralytics was trained on the randomly selected X-ray_split_1 dataset with default parameters. A detailed list of all the default hyperparameters and augmentation settings can be found at [40]. Throughout the study, we considered mAP@50–95 > 0.5 for the majority of single-cell classes, mAP@50 > 0.5 for filamentous classes, and overall mAP@50–95 > 0.5 as the criteria for a well-performing model. Moreover, validation metrics, including class loss, box loss, and distribution focal loss (dfl), were monitored to prevent overfitting and training instability. All metrics and loss functions were generated by the Ultralytics framework [40].

### Hyperparameter optimization

A hyperparameter search was performed using the hyperparameters obtained from the exploratory search (see Table S2). For tuning the hyperparameters of the YOLOv8x model on our custom dataset, the “model.tune()” method was used. This utilised the “Tuner” class based on the Ultralytics YOLO mutation algorithm to search for the best-fit hyperparameters [41]. A total of 45 iterations were executed, each with a limit of 300 epochs, using the Stochastic Gradient Descent (SGD) optimiser.

### Cross-validation runs

Using the tuned hyperparameters (see Table S3), we trained models on all five stratified folds for each dataset family. This procedure was applied to the X-ray and Yankee datasets, whereas for the Zulu dataset family, augmentation parameters were slightly modified to provide more robust augmentation; the complete parameter sets are listed in Tables S3 and S4, respectively.

### Performance evaluation

The model’s performance was evaluated using the standard metrics- Intersection over Union (IoU), box-level precision (P) and recall (R), and mean average precision (mAP). The mAP was computed at an IoU threshold of 0.50 (mAP@50) and averaged values across varying thresholds from 0.50 to 0.95 with an increment of 0.05, mAP@50–95. During training, three loss functions were monitored: classification loss, box loss, and distribution focal loss (dfl). F1 score curves, precision-recall (PR) curves, raw and normalised confusion matrices, all generated by the Ultralytics framework, were also evaluated [42].

## Graphical user interface and data analysis suite

A cross-platform GUI was implemented using PyQt5 [43]. All image handling operations were performed using Pillow (v11.2.1) [44] and OpenCV [45], including image dimension capture, resizing, and rendering. The prediction script was integrated into the GUI, allowing parameterization through interface controls. Except for the imgsz parameter (automatically determined from image dimensions) and interface-based inputs, all model parameters were set to their default values. Annotation data was extracted and analysed to quantify predicted morphotypes.

### Edge bounding box exclusion

A key data preprocessing step excluded bounding boxes at the edges of images to avoid counting partially visible cells (false positives or incorrect detections). Bounding boxes were excluded from the analysis if either the left or right edges (calculated as centre x ± width/2), or the top or bottom edges (centre y ± height/2), were within a small threshold (epsilon = 0.001) of the image border (0 or 1 in normalised coordinates). This feature was deactivated for classes C6, C7-E, C7-F, and C7-G.

### Plotting

All numerical operations were performed on “.txt” files exported by the Ultralytics framework output using standard NumPy & SciPy libraries [46, 47]. For rendering interactive visualisations, including absolute-count bar plots, relative-abundance bar plots, mean relative-abundance bar plots, normalised stacked bar plots, and hierarchical heatmaps (Ward’s method), the Plotly library (v6.1.2) [48] was used. The data analysis pipeline operated in two modes: single-group and multi-group. In both modes, three summary metrics were computed for each morphotype class. Absolute counts represented the total number of detections per class summed across all images. Relative abundance (%) represented the contribution of each class to the total number of detections across all images in the dataset or group. Mean relative abundance (%) was computed by first expressing each class as a fraction of the total detections within each individual image and then averaging these per-image fractions across all images, such that each image contributed equally to the final value regardless of how many cells were detected in it. Morphotype composition per image or per group was additionally displayed as normalised stacked bar plots, where each bar summed to 100% and each segment represented the proportional contribution of one morphotype. Hierarchical clustering heatmaps were generated from per-image, per-group relative abundance values using Ward’s linkage method on Euclidean distances, with the resulting leaf order applied to reorder the image or group axis of the heatmap.

## Results and discussion

### Morphological assessment and class definitions

**Fig. 2:**
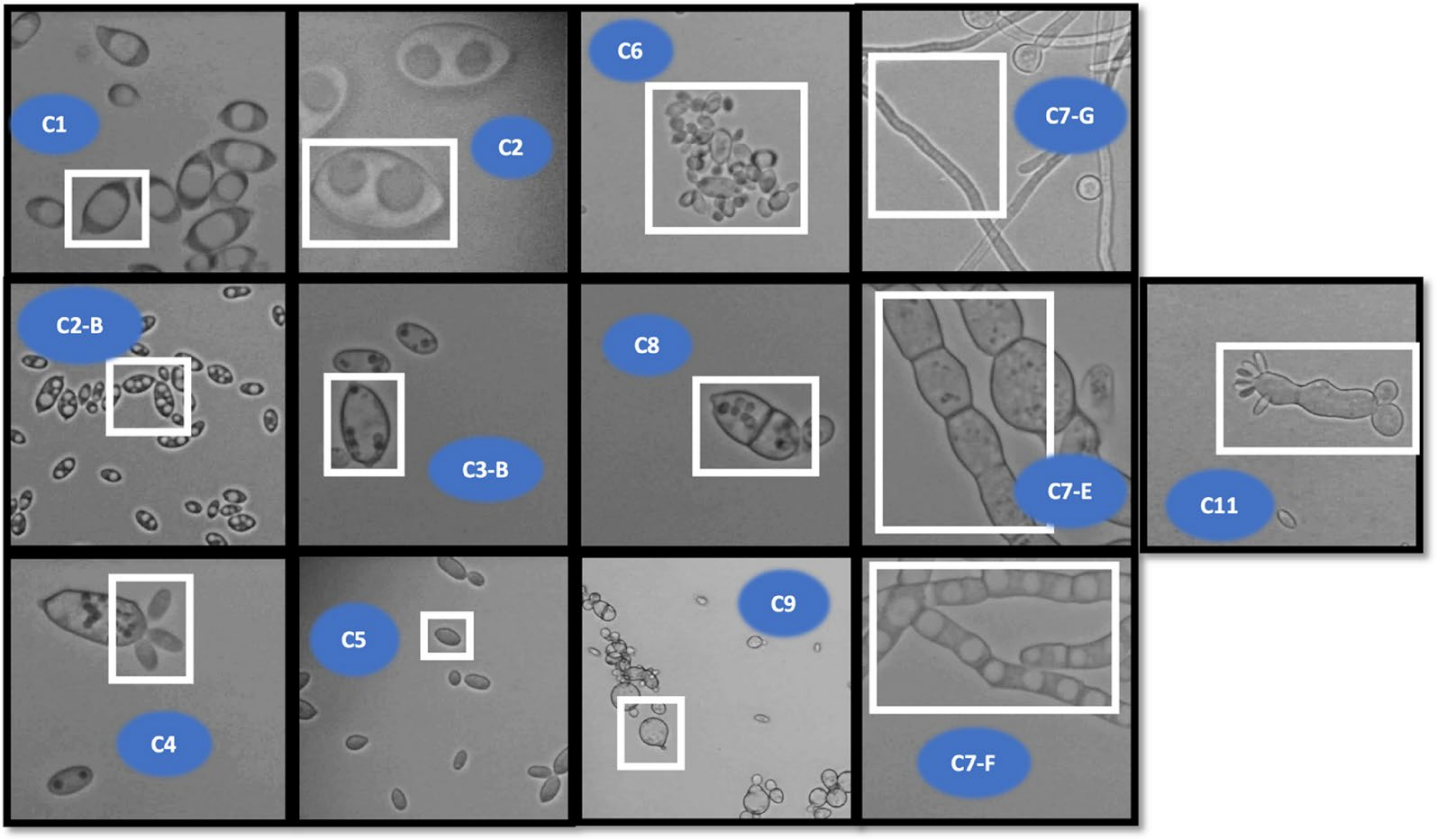
TU_MyCo-Vision classes and representative cell morphologies, highlighted with white rectangular boxes. C1, C2, and C2-B are vacuolated cells. C3-B are cells with granular cytoplasm. C5 are general yeast-like cells lacking any special features. C9 cells are spheroidal-shaped cells. C4, C6, and C11 are special cell types representing budding events, cell clumping/aggregates, and irregular cell shapes. C7-E (septate hyphae/ pseudo hyphae), C7-G (smooth continuous filamentous/true hyphae), and C7-F (vacuolated hyphae) are filamentous cell classes

Based on the observations from the brightfield microscopic images from *A. pullulans*,* A. melanogenum*, and *T. reesei cultivations*, cells were empirically distributed into thirteen morphotype classes, based on their distinct biological features like cell shape, vacuolation, granularity of cytoplasm, presence of septa, budding, clumping and filament type (hyphae, pseudohyphae, vacuolated hyphae) (Fig. 2). Classes were grouped into single-cell morphologies (C1, C2, C2-B, C3-B, C5, C9), filamentous morphologies (C7-E, C7-F, C7-G), and special morphologies (C4, C6, C8, C11). Refer to Table 1 for detailed descriptions of each class.

**Table 1:** Class description of TU_MyCo-Vision object classes

Class	Description	Defining features
C1	Vacuolated single cells (eye-like appearance)	Ovoid, oblong, spheroidal or elongated single cells containing a single, prominent vacuole, giving an eye or egg-like appearance.
C2	Vacuolated single cells with prominent binary vacuoles	Ovoid, oblong, spheroidal or elongated single cells with two distinct vacuoles, producing a characteristic binocular appearance.
C2-B	Heavily vacuolated single cells	Ovoid, oblong, spheroidal or elongated single cells containing multiple vacuoles distributed within the cytoplasm.
C3-B	Single cells with granular cytoplasm	Ovoid, oblong, spheroidal or elongated single cells with a granular cytoplasm characterized by numerous small bead-like structures.
C4	Budding events	Objects representing active budding, including mother–bud pairs, and bud emergence on hyphae.
C5	Yeast-like cells	Ovoid, oblong or elongated single cells without distinctive vacuoles, granules, septa, or irregularities, encompassing general yeast-like cells that lacked any distinctive features.
C6	Cell aggregates and clumps	Multicellular aggregates or clumps of cells that could not be reliably segmented into individual objects, including dense clusters and irregular aggregates.
C7-E	Septate filaments (Septate hyphae/pseudohyphae)	Filamentous structures with an appearance consistent with septate hyphae or pseudohyphae, characterised by visible transverse septa and elongated, often branching morphology; vacuolation and granularity were variable and not used as primary criteria.
C7-F	Vacuolated filaments	Filamentous structures containing vacuoles along their length, often appearing as discrete vacuolar compartments within the hyphal segment; septa could be present or absent, but vacuolation was the defining feature.
C7-G	Smooth filaments (non-septate / true hyphae)	Smooth, continuous filamentous structures lacking a clearly resolved septa in brightfield images; these were interpreted as non-septate hyphae and distinguished from C7-E and C7-F by their smooth and uniform appearance; internal granularity could be variable.
C8	Septate cells	Non-filamentous, often enlarged single cells or short segments displaying one or more internal septa, without forming extended filaments; vacuole content and cytoplasmic granules could vary, but the presence of clear septation in an otherwise cell-like unit was the decision criterion.
C9	Spheroidal cells	The main criterion was the spherical-to-spheroidal geometry of the cell, typically more isotropic than elongated yeast-like cells or other single-cell morphologies described; these were separated from C5 based on near-spherical geometry.
C11	Irregularly shaped cells	Single cells with irregular, non-ovoid outlines that could not be assigned to other single-cell classes, including highly deformed, angular, or swollen morphologies; vacuoles, granules, or septa could be present but were not consistently interpretable across instances.

## Generation of the first dataset “X-ray”, exploratory training, and hyperparameter optimisation

The first version of our image dataset “X-ray” comprised 5 dataset splits from 5-fold stratified splitting, with 1,028 manually annotated images (80:20, Training: validation split) containing 8,575 annotated objects (cells), combining the training and validation sets across the 13 defined classes. Across the five training splits, the average object count was 6,860 cells; across the validation sets, it was 1,715 cells. The class distribution was markedly uneven: C5 (yeast-like cells) was by far the most represented class, contributing on average 2,370.4 (SD 15.36) objects in all five splits. Class C5 was followed by C1 in terms of its abundance; the average number of objects was 936 (SD ± 50.53) across training splits. C2-B (heavily vacuolated cells) and all filamentous cell classes (C7-E, C7-F & C7-G) were underrepresented. Containing 351.2 (SD ± 17.97), 190.2 (SD ± 4.50), 262.4 (SD ± 6.37) & 338.4 (SD ± 8.16) objects on average, respectively, across all five training splits. C11 & C8 were the least abundant classes, with average numbers of objects of 72 (SD 2.68) and 58.4 (SD 1.85), respectively. Despite this imbalance, stratified splitting ensured that the relative proportions of each class were preserved across all five folds, and the validation dataset also reflects the same distribution, as confirmed by the similar standard deviations for each class in both the training and validation datasets. For detailed object distribution metrics, refer to Table S5.

### Exploratory training

Initial training used the YOLOv8x architecture on the split_1 subset of the X-ray dataset, chosen as a representative fold from the stratified 5-fold cross-validation dataset. Using default Ultralytics hyperparameters as a starting point, we performed an exploratory search to identify a reasonable configuration (Table S2), which yielded satisfactory early-stage performance across multiple fungal morphotypes, particularly for the major single-cell classes.

In this Version_1 model, detection accuracy was highest for predominant single-cell morphotypes. The mAP@50 values were 0.840 for C1 (cells with a central vacuole), 0.816 for C2 (dual vacuoles), 0.785 for C5 (yeast-like morphologies), 0.822 for C2-B (multiple vacuoles), 0.904 for C3-B (granular cytoplasm), and 0.890 for C8 (septate cells), indicating that spheroidal and ovoid cells with clear vacuolar or septation patterns are readily captured by the detector. In contrast, filamentous morphologies remained more challenging, with mAP@50 values of 0.811, 0.566, and 0.547 for C7-E (septate hyphae or pseudohyphae), C7-F (vacuolated hyphae), and C7-G (smooth/non-septate hyphae), respectively, underscoring the greater complexity of hyphal pattern recognition compared with single-cell forms.

The normalized confusion matrix (Supplementary Folder S7) revealed systematic misclassification among geometrically similar single-cell classes. Class C5 (yeast-like cells) was particularly affected and was frequently predicted as C11 (irregular shapes) and C9 (spheroidal cells). Although 80% of ground-truth C5 instances were correctly identified, the class exhibited a 36% hallucination rate (proportion of predicted C5 objects without a corresponding ground-truth instance). These patterns highlight the challenges due to the intrinsic biological and geometric overlap between yeast-like and spheroidal morphologies and illustrate the difficulty of cleanly separating closely related single-cell states in brightfield images.

Across training epochs, the highest mAP@50–95 of 0.5216 was achieved at epoch 486, meeting our predefined threshold for a functional prototype. The corresponding hyperparameter configuration was therefore selected as the baseline for subsequent optimisation. Detailed performance metrics, including precision–recall curves, F1 curves, and confusion matrices, are provided in the Supplementary Folder S7.

### Hyperparameter optimisation

We used the Ultralytics genetic mutation-based hyperparameter tuner [41], which iteratively adapts training parameters to maximise the fitness score. The best configuration emerged at iteration 19 with a fitness score of 0.5491, yielding a precision of 73.21%, a recall of 68.01%, an mAP@50 of 0.7268, and mAP@50–95 of 0.5293, indicating an improvement over the exploratory baseline. Fitness values plateaued after iteration 19, with the second-best score (0.5404) observed at iteration 23 (Supplementary Fig. 1), indicating no benefit from further mutation cycles. The optimised hyperparameters (Table S3) were subsequently applied to all training runs.

Hyperparameter optimisation was an important component of our transfer-learning pipeline, because hyperparameters derived from generic benchmarks are often suboptimal for domain-specific, multi-class object-detection tasks such as fungal morphotype recognition. Given the pronounced class imbalance in our dataset, explicit hyperparameter tuning was an important step in model development. In imbalanced datasets, the interplay between learning rate, regularisation, loss weighting, and sampling strategy can strongly bias a model toward overrepresented classes at the expense of sensitivity to rare morphotypes. Hyperparameter tuning with optimised class weights has been shown to improve classifier performance regardless of the algorithm or encoding strategy used, consistently outperforming both native downsampling, which discards potentially informative instances from majority classes, and naive upsampling, which risks overfitting by duplicating minority-class examples. These considerations motivated our use of systematic hyperparameter optimisation rather than relying on default training configurations [49]. (see Supplementary Folder S8 “hyperparameter tuning” for additional details).

## X-ray - The first trained model

As part of the 5-fold cross-validation strategy, a separate model was trained on each of the training dataset splits. The models were evaluated using the standard object detection metrics, including box precision, box recall, mAP@50 and mAP@50–95. The performance criteria were as described in the methods : mAP@50–95 > 0.5 for the majority of single-cell classes and mAP@50 > 0.5 for filamentous classes, as the criteria for a well-performing model.

In the YOLO framework, mAP@50 denotes the mean Average Precision computed at a single Intersection over Union (IoU) threshold of 0.5, meaning a predicted bounding box is counted as correct if its overlap with the ground truth is at least 50%, and the final score is averaged over all classes as the area under the precision–recall curve at this IoU [50]. By contrast, mAP@50–95 is a stricter COCO-style metric that averages the AP values over multiple IoU thresholds from 0.50 to 0.95 in steps of 0.05, thereby rewarding models that maintain both correct detection and precise localisation across a wide range of overlap criteria [51]. In both cases, mAP values range from 0 to 1 (0–100%), where values closer to 1 indicate that the detector consistently achieves high precision and recall across the evaluated classes (morphotypes) or overall performance.

Validation metrics, including class loss, box loss, and distribution focal loss, were monitored for overfitting and training instability. Along with these metrics, F1 score curves, precision-recall (PR) curves, and raw and normalised confusion matrices were also evaluated. This similar setup was used for all future evaluations.

For ease of readability and interpretation, this section discusses only the best-performing model. For detailed split-wise metrics, refer to the Supplementary Folder S9 (X-ray).

The X-ray split_4 model achieved good classification accuracy (i.e., all detected objects were assigned to their correct morphological class, according to the normalized confusion matrix) for common morphotypes, including C1 (89%), C2 (88%), C5 (84%), C6 (90%), C8 (88%), and C2-B (79%), with moderate performance for C3-B (76%), C4 (73%), and C11 (58%). C9 was misclassified as C5 21% of the time, and misclassification of C1 primarily occurred with C2 and C2-B, at 4% and 6%, respectively. It is to be noted that C1, C2 and C2-B are all variations of vacuolated morphologies.

Filamentous classes were particularly challenging: C7-E, C7-F, and C7-G were correctly predicted in 75%, 58%, and 52% of cases, respectively, with 20–45% of bounding boxes assigned to the background, indicating that the model hallucinated and detected filamentous morphology even when it was absent. Based on the Precision Recall curve, corresponding mAP@50 values were 0.649, 0.440, and 0.429, respectively, while higher scores were observed for C1 (0.914), C2 (0.902), C2-B (0.939), C6 (0.921), and C8 (0.882).

Thus, except for the C7-E (Sepate or pseudo-hypahe), the performance of the model for C7-F (vacuolate hyphae), C7-G (smooth hyphae) was subpar to our set standards. However, closer inspection of the class-wise object counts and performance metrics did not reveal a simple monotonic relationship between the number of objects in a given class and its performance. This suggests that the optimised training configuration mitigated, but did not necessarily eliminate, a straightforward majority-class bias, and points to a reasonably balanced yet still class-dependent training.

## Transition to the Yankee dataset for performance improvements

The Yankee dataset reflects a series of curatorial and annotation decisions aimed at increasing the object counts for some difficult-to-detect classes and cleaner annotations. The most biologically meaningful change is the marked increase in C1 (vacuolated single cells with a central vacuole), which rose from a mean of 936.0 to 1199.2 cells per training split (+ 28.1%). This gain resulted from annotating individual C1 cells that had previously been kept unannotated when contained within a clump/aggregate. This was performed with the possibility in mind that vacuolated cell clumping may reflect an underlying biological phenomenon that the model should not miss. This was done not only for C1, but also for other morphologies except C5 (Normal yeast-like cells), as it would inflate the already high count of these cells in the dataset.

Conversely, the dominant C5 class (yeast-like cells) was reduced from 2,370.4 to 2,176.8 objects per split (− 8.2%), as ambiguously labelled or poorly focused instances were removed during image review. For the filamentous morphotypes C7-E (septate filaments), C7-F (vacuolated filaments), and C7-G (smooth filaments), the revised annotation strategy replaced multiple overlapping bounding boxes over large hyphal regions with fewer, spatially broader boxes; object counts therefore changed minimally (within ± 2.5%), while biological coverage was preserved or improved. The rarest morphotypes, C8 (septate cells) and C11 (irregularly shaped cells), remained essentially unchanged, underscoring that annotation refinement alone is insufficient to address severe class imbalance; enrichment of these categories requires imaging samples with naturally higher abundance of these forms Table 2.

**Table 2:** Mean object counts per cross-validation split (*n* = 5) for X-ray and Yankee datasets

Class	Morphotype	X-ray train mean (SD)	Yankee train mean (SD)	Δ objects	Δ %	Yankee val mean (SD)
C5	Yeast-like cells	2370.4 (15.36)	2176.8 (62.08)	−193.6	−8.2%	544.2 (62.08)
C1	Vacuolated single cells	936.0 (50.53)	1199.2 (56.22)	+ 263.2	+ 28.1%	299.8 (56.22)
C9	Spheroidal cells	560.0 (12.99)	630.4 (28.20)	+ 70.4	+ 12.6%	157.6 (28.20)
C4	Budding events	472.0 (16.46)	518.4 (20.86)	+ 46.4	+ 9.8%	129.6 (20.86)
C3-B	Granular cytoplasm cells	444.8 (26.86)	394.4 (12.63)	−50.4	−11.3%	98.6 (12.63)
C2	Dual-vacuole cells	405.6 (12.88)	400.0 (14.35)	−5.6	−1.4%	100.0 (14.35)
C6	Cell aggregates / clumps	398.4 (6.56)	343.2 (7.19)	−55.2	−13.9%	85.8 (7.19)
C2-B	Heavily vacuolated cells	351.2 (17.97)	340.0 (13.99)	−11.2	−3.2%	85.0 (13.99)
C7-G	Smooth filaments	338.4 (8.16)	338.4 (1.62)	0.0	0.0%	84.6 (1.62)
C7-F	Vacuolated filaments	262.4 (6.37)	268.0 (7.62)	+ 5.6	+ 2.1%	67.0 (7.62)
C7-E	Septate filaments	190.4 (4.50)	185.6 (4.76)	−4.8	−2.5%	46.4 (4.76)
C11	Irregularly shaped cells	72.0 (2.68)	69.6 (4.45)	−2.4	−3.3%	17.4 (4.45)
C8	Septate cells	58.4 (1.85)	57.6 (3.14)	−0.8	−1.4%	14.4 (3.14)

SD = population standard deviation. Δ = change from X-ray to Yankee training set. Validation SD mirrors training SD by design of stratified splitting

### Training of the yankee model

The Yankee split_5 model showed improvements over X-ray split_4 across several classes, most notably for C7-E (septate hyphae/pseudohyphae), whose mAP@50 increased from 0.649 to 0.756 and classification accuracy from 75% to 85%, the largest single-class gain between the two model families, and could be a consequence of replacing overlapping filamentous bounding boxes with fewer, larger annotations that provided a cleaner training signal. Classification accuracy also improved or remained stable for the majority of single-cell classes, including C1 (89%), C2 (82%), C3-B (80%), C5 (80%), C6 (85%), and C8 (80%). The overall mAP@50 increased modestly from 0.760 to 0.766.

Despite these gains, C7-F (vacuolated filaments) and C7-G (smooth filaments) remained the weakest classes, with mAP@50 values of 0.592 and 0.500, respectively, but above the X-ray split_4 values of 0.440 and 0.429. This is not only a notable improvement over the previous model, but, more importantly, the mAP@50 for filamentous classes was at or above our set threshold.

Persistent misclassification between geometrically similar single-cell classes, C9 (spherical cells), confused with C5 (yeast-like cells) in 18% of cases, and C3-B (ovoid cells with granulated cytoplasm) with C5 in 14% cases, further reflects the inherent difficulty of separating geometrically closely related morphotypes. Collectively, these results confirmed that while the Yankee curation substantially improved detection for filamentous classes and overall performance, the underrepresentation of C7-F, C7-G, C8, and C11 remained unresolved, providing the primary motivation for an expanded, representative dataset & model, especially for difficult-to-detect morphologies. Refer to Supplementary Fig. 2 for the PR curve and confusion matrix of X-ray and Yankee models. For detailed split-wise metrics, refer to the Supplementary Folder S11 (yankee).

## Hybrid dataset – Zulu

The transition from the Yankee to the Zulu dataset was achieved by combining manually annotated images from the Yankee dataset with automatically labelled images. Specifically, we used the Yankee_s5 model to generate labels for previously unused images from our image bank, treated these predictions as annotations after quality control, added the newly labelled images to the existing Yankee dataset, and used them to train a new model. In total, 14,200 images were annotated, of which only 516 high-quality images were included in the dataset after manual review, based on the previously described annotation and QC strategy. Curation efforts were directed towards the morphotype classes that proved most difficult for the model to learn in earlier training runs, as well as towards general class-balancing objectives. Overall, the mean number of annotated objects per training split increased from 6,921.6 in Yankee to 9,443.2 in Zulu, a gain of 2,521.6 objects per fold. Table 3 summarises the per-class changes.

The most pronounced gains were recorded for filamentous morphologies. Smooth filaments (C7-G) nearly doubled in representation (+ 96.22%), while heavily vacuolated cells (C2-B) and granular cytoplasm cells (C3-B) increased by + 70.12% and + 68.15%, respectively. Dual-vacuole cells (C2) also grew substantially (+ 51.60%). These targeted additions reflect efforts to increase the dataset’s total number of objects and images and to achieve class balance.

By contrast, the two rarest classes in terms of number of objects (cells), septate cells (C8) and irregular cells (C11), remained the least represented in Zulu despite curation efforts, with only modest absolute gains of 4.0 and 5.6 objects per split, respectively. This persistent scarcity reflects a genuine biological constraint: these morphotypes are rarely observed in routine culture conditions, limiting the volume of images available for annotation regardless of effort. Refer to Supplementary Fig. 3 for the PR curve and confusion matrix of Yankee and Zulu models.

**Table 3:** Mean object counts per cross-validation split (*n* = 5) for Yankee and Zulu datasets

Class	Morphotype	Yankee train mean (SD)	Zulu train mean (SD)	Δ objects	Δ %	Zulu val mean (SD)
C5	Yeast-like cells	2176.8 (62.08)	2762.4 (5.78)	+ 585.6	+ 26.9%	690.6 (5.78)
C1	Vacuolated single cells	1199.2 (56.22)	1614.4 (35.27)	+ 415.2	+ 34.6%	403.6 (35.27)
C9	Spheroidal cells	630.4 (28.20)	804.8 (26.50)	+ 174.4	+ 27.7%	201.2 (26.50)
C4	Budding events	518.4 (20.86)	653.6 (27.97)	+ 135.2	+ 26.1%	163.4 (27.97)
C3-B	Granular cytoplasm cells	394.4 (12.63)	663.2 (15.35)	+ 268.8	+ 68.2%	165.8 (15.35)
C2	Dual-vacuole cells	400.0 (14.35)	606.4 (20.98)	+ 206.4	+ 51.6%	151.6 (20.98)
C6	Cell aggregates/clumps	343.2 (7.19)	410.4 (12.03)	+ 67.2	+ 19.6%	102.6 (12.03)
C2-B	Heavily vacuolated cells	340.0 (13.99)	578.4 (6.83)	+ 238.4	+ 70.1%	144.6 (6.83)
C7-G	Smooth filaments	338.4 (1.62)	664.0 (7.07)	+ 325.6	+ 96.2%	166.0 (7.07)
C7-F	Vacuolated filaments	268.0 (7.62)	305.6 (4.76)	+ 37.6	+ 14.0%	76.4 (4.76)
C7-E	Septate filaments / pseudohyphae	185.6 (4.76)	243.2 (5.27)	+ 57.6	+ 31.0%	60.8 (5.27)
C11	Irregular cells	69.6 (4.45)	75.2 (4.79)	+ 5.6	+ 8.0%	18.8 (4.79)
C8	Septate cells	57.6 (3.14)	61.6 (5.89)	+ 4.0	+ 6.9%	15.4 (5.89)

SD = population standard deviation. Δ = change from Yankee to Zulu training set. + = increase; − = decrease. Validation SD mirrors training SD by design of stratified splitting

### Training of model Zulu

Training on the expanded Zulu dataset yielded clear and meaningful gains across the morphological classes. In the best performing model, Zulu_Split1, among single-cell classes, vacuolated cells (C1) reached 95% classification accuracy (mAP@50 0.963), and spheroidal cells (C9) improved from 61% to 72%. Detection of granular cytoplasm cells (C3-B) also improved, reaching 84% accuracy (mAP@50 0.924). In comparison, cell aggregates or clumps (C6) rose from 85% to 89%, gains that likely reflect a more consistent representation of these morphotypes across the expanded image set. Budding events (C4) and irregular cells (C11) remained the most challenging classes, a result consistent with the inherent annotation difficulty of transitional or heterogeneous morphologies rather than a model artefact. The most deliberate enrichment effort targeted filamentous classes: smooth hyphae (C7-G), whose training object count nearly doubled, improving from 51% to 67% accuracy, while vacuolated hyphae (C7-F) rose from 55% to 71%, improvements that directly translate into better detection of filamentous morphologies.

These gains were not captured by any single metric; for example, C7-E showed a modest decline in on-diagonal accuracy while increasing mAP@50 (0.756 → 0.808), underscoring the importance of evaluating multiple performance dimensions, mAP@50, mAP@50–95, precision–recall curves, and confusion matrices for performance assessment. Taken together, overall mAP@50 rose from 0.766 (Yankee) to 0.789 (Zulu), with gains distributed across both common and rare morphotypes, confirming that iterative, targeted dataset refinement translates into measurable improvements in the detection of biologically relevant fungal morphologies.

Across all three iterative datasets, model performance improved progressively with each cycle of dataset refinement (Table 4). Mean precision increased from 71.6% (X-ray) to 74.6% (Zulu), mAP@50 from 0.744 to 0.775, and mAP@50–95 from 0.536 to 0.573, while the dataset grew from 1,028 to 1,504 images. The largest single-step gains occurred between Yankee and Zulu, coinciding with the most deliberate curation effort. Recall remained stable across all generations (71.8–73.0%). Taken together, these results suggest that targeted, class-centric dataset curation contributed to improving the model performance. For detailed split-wise metrics, refer to the Supplementary Folder S10 (zulu).

**Table 4:** Comparative summary of average performance metrics across five dataset splits of X-ray, Yankee, and Zulu model families evaluated on their validation dataset

Metric	X-ray	Yankee	Zulu
Dataset version (No. of images)	1,028	1,002	1,504 (Manual + auto labelled)
Architecture	YOLOv8x	YOLOv11m	YOLOv11m
Best Split	xray_split4	Yankee_split5	Zulu_split1
Highest mAP@50–95 at epoch	309	405	483
Precision (mean ± SD)	71.56 ± 0.0174	72.43 ± 0.0160	74.59 ± 0.0230
Recall (mean ± SD)	71.83 ± 0.0356	71.67 ± 0.0203	72.97 ± 0.0163
mAP@50 (mean ± SD)	0.7443 ± 0.0202	0.7535 ± 0.0147	0.7745 ± 0.0106
mAP@50–95 (mean ± SD)	0.5360 ± 0.0127	0.5357 ± 0.0096	0.5732 ± 0.0123

## Cross-validation and performance evaluation of all model families

To obtain comparable performance estimates across different model families. A separate global test dataset of 166 images was created (Refer to Table S6, for object distribution in global test set), and all 15 models (5 per family) were validated on it. Refer to Supplementary Folder S13 (validation_global_set) for detailed performance metrics of all models. The evaluation criteria were consistent with all previous evaluations, but for numerical comparison, emphasis was placed on mAP@50 and mAP@50–95. When the mAP values were comparable, precision was used as a tiebreaker.

This dataset provides a stricter evaluation by eliminating performance differences caused by variations in validation datasets. Even though the distribution of objects is similar across splits due to the stratified 5-fold strategy, the complexity of images or objects can vary; e.g., some images may contain multiple regions with difficult-to-detect hyphae, while others may have fewer complex filaments and regions. This could be an important cause of variation in performance across splits within a dataset family.

Across all three model families, detection performance improved progressively with each dataset iteration, consistent with the incremental improvements in image quality, annotation consistency, and class balance described in the Methods. This observation is consistent with the model performance on the validation dataset. X-ray models achieved mAP@50–95 values of 0.493–0.513 and mAP@50 of 0.648–0.672. Yankee models showed moderate improvement, with mAP@50–95 ranging from 0.498 to 0.526 and mAP@50 from 0.658 to 0.709. Zulu models performed best overall, reaching mAP@50–95 values of 0.519–0.546 and mAP@50 of 0.689–0.735 (Table 5).

**Table 5:** Precision, Recall, and mAP@50–95 performance comparison of all TU_MyCo-Vision model families on the global test dataset (s = split)

Model	Precision	Recall	mAP@50–95
xray_s1	0.708	0.65	0.496
xray_s2	0.64	0.661	0.511
xray_s3	0.691	0.604	0.513
xray_s4	0.631	0.649	0.493
xray_s5	0.64	0.648	0.494
Yankee_s1	0.659	0.649	0.505
Yankee_s2	0.619	0.68	0.498
Yankee_s3	0.653	0.676	0.518
Yankee_s4	0.705	0.661	0.521
Yankee_s5	0.686	0.684	0.526
Zulu_s1	0.658	0.664	0.521
Zulu_s2	0.715	0.612	0.519
Zulu_s3	0.734	0.665	0.545
Zulu_s4	0.638	0.717	0.527
Zulu_s5	0.715	0.679	0.546

Within the Zulu family, Zulu_s5 achieved the highest mAP@50–95 (0.546), and Zulu_s3 reached the highest precision (73.4%) and mAP@50 (0.735), with mAP@50–95 of 0.545. Given the negligible difference in mAP@50–95 between Zulu_s5 and Zulu_s3 (0.001) and Zulu_s3’s higher precision and mAP@50, we selected Zulu_s3 as the representative model for TU_MyCo-Vision. In the context of morphotype quantification, where false-positive detections directly inflate morphotype counts in a culture, precision is a particularly important criterion, and Zulu_s3 offers the best balance of localisation accuracy and detection reliability across the full morphotype spectrum.

Examining the per-class performance of Zulu_s3 in detail (Table 6) reveals a biologically interpretable pattern of strengths and limitations. Detection was strongest for morphotypes with clear, distinctive brightfield features: C3-B (cells with granular cytoplasm; mAP@50 0.900%, recall 94.9%), C2 (cells with dual vacuoles; mAP@50 91.2%, recall 85.1%), C2-B (heavily vacuolated cells; mAP@50 88.7%), C8 (septate cells; mAP@50 0.897, precision 90.0%), and C1 (single-vacuole cells; mAP@50 0.844) all achieved strong performance. These classes share well-defined intracellular structures, vacuoles, septa, or granular inclusions, that are consistently resolved under standard brightfield optics and therefore provide reliable anchors for the detector.

Yeast-like cells without distinctive features (C5; mAP@50 0.792, recall 78.9%) and spheroidal cells (C9; mAP@50 0.583) were detected adequately, though the normalised confusion matrix reveals systematic misclassification between these geometrically overlapping classes. Specifically, 14% of C1 cells were misclassified as C5, 14% of C2-B cells as C5, and 11% of C9 cells as either C5 or C3-B, illustrating the challenge of resolving morphotypes that differ primarily in intracellular content (vacuoles, granules) rather than overall cell shape. In brightfield images, subtle differences in vacuolation state or cytoplasmic texture can appear ambiguous, particularly at lower magnifications or in dense fields, a limitation that is inherent to the imaging modality rather than the model architecture.

Filamentous morphotypes presented the greatest detection challenge, as expected given their structural complexity, variable extension across the image field, and the difficulty of defining consistent bounding box boundaries for objects that often span a significant fraction of the 640 × 640 px tile. C7-F (vacuolated hyphae) achieved the highest performance among filamentous classes (mAP@50 of 0.712, precision 72.0%), benefiting from the distinctive vacuolar compartments along the hyphal length. C7-E (septate hyphae/pseudohyphae; mAP@50 0.577) and C7-G (smooth, non-septate hyphae; mAP@50 0.517) showed lower but still meaningful detection rates, with classification accuracies of 57% and 58% in the confusion matrix, respectively. In practical terms, these values indicate that the model can reliably signal the *presence* of filamentous growth in an image at least 50% of the time, and the detection of hyphal classes should be interpreted as a presence/absence or a semi-quantitative indicator rather than a precise count. For budding events (C4; mAP@50 of 0.622), recall was particularly limited (47.5%). Cell aggregates (C6; mAP@50 0.699) and irregularly shaped cells (C11; mAP@50 0.612) performed at intermediate levels; C11, in particular, remains the most challenging class due to its definition as a morphological catch-all for cells that do not fit the primary categories, resulting in high intra-class variability.

**Table 6:** Class-wise performance metrics of the Zulu_s3 model evaluated on the global test dataset

Class	Images	Instances	Precision	Recall	mAP@50	mAP@50–95
all	166	956	0.734	0.665	0.735	0.545
C2	27	55	0.839	0.851	0.912	0.730
C5	77	229	0.709	0.789	0.792	0.587
C1	31	78	0.762	0.731	0.844	0.677
C4	63	139	0.811	0.475	0.622	0.327
C6	16	25	0.639	0.600	0.699	0.560
C11	8	12	0.613	0.583	0.612	0.480
C9	27	38	0.557	0.579	0.583	0.459
C2-B	28	95	0.865	0.739	0.887	0.729
C3-B	36	74	0.778	0.949	0.900	0.730
C8	20	26	0.900	0.846	0.897	0.790
C7-E	36	65	0.664	0.446	0.577	0.338
C7-F	32	49	0.720	0.571	0.712	0.391
C7-G	30	71	0.680	0.479	0.517	0.291

Taken together, these results indicate that Zulu_s3 provides biologically interpretable morphotype detection for the majority of the thirteen defined classes, with performance characteristics appropriate for population-level morphotype quantification in *Aureobasidium* cultures. The model is most informative for distinguishing yeast-like cells, vacuolated, granular, and septate single-cell morphologies, while filamentous and morphologically ambiguous classes should be interpreted with appropriate caution. Detailed per-class metrics and performance visualisations are provided in Table S7 and Supplementary Folder S13, respectively.

## Graphical user interface and data analysis suite

### Graphical user interface and integrated data analysis

We implemented a cross-platform graphical user interface (GUI) to make TU_MyCo-Vision accessible. All dependencies and libraries are packaged into single executables for Windows (.exe) and macOS (.app), which is a good alternative to manual environment setup. The GUI is organised into three main panels: Detection, Analysis, and Results Dashboard.

- The Detection panel handles input/output folder selection, morphotype class selection, model parameter settings, and prediction execution (Supplementary Fig. 4).
- The Analysis panel provides single-group and multi-group analysis, including a class-exclusion module that can group selected morphotypes into an “Others” category (e.g. grouping filamentous classes C7-E/F/G).
- The Results Dashboard offers side-by-side visualization of original and annotated images and an embedded CSV viewer. It can render interactive plots (relative abundance, normalised stacked bars, heatmaps) directly from the loaded CSV via a “Click to View Plots” control (Supplementary Fig. 4b–d).

This design links object detection with quantitative morphology readouts in a single environment.

### Validation of single and multi group data analysis pipeline and exploratory morphotype profiling across three fungal Genera

#### Single-group analysis: pipeline validation on the global test set

To confirm that the data processing and visualisation pipeline functions correctly and produces biologically interpretable outputs, we applied the single-group analysis workflow to 90 images drawn from the independent global test set. Two parallel workflows were evaluated: one using all 13 morphotype classes, and a second in which the three filamentous classes (C7-E, C7-F, and C7-G) were consolidated into a single “Others” category using the class-exclusion module. This grouping reflects a common biological scenario in which the investigator wishes to distinguish unicellular from filamentous growth states without resolving filament subtypes, for example, when assessing the transition from yeast-like to hyphal growth at the population level.

In both workflows, the pipeline successfully processed all 90 images. It generated the complete set of six quantitative visualisations: absolute-count bar plots, normalised stacked bar plots, relative abundance plots, mean relative abundance plots, and hierarchical clustering heatmaps (Supplementary Figs. 5–6). The normalised stacked bar plots revealed the expected diversity of morphotype compositions across images, with vacuolated single-cell classes (C1, C2, C2-B) and yeast-like cells (C5) dominating most fields. After applying class exclusion, the “Others” category, all filamentous morphologies were grouped into the “Other” category, and the remaining unicellular classes showed comparable relative abundances to the unfiltered workflow, confirming that consolidation does not distort the underlying morphotype distribution. But aids in interpreting the experimental outcomes. Taken together, these results demonstrate that TU_MyCo-Vision can summarise morphotype distributions at scale, and that biologically motivated class groupings can be applied flexibly through the GUI.

#### Multi-group analysis: exploratory morphotype profiling in genera outside the training set

A central question for the practical utility of TU_MyCo-Vision is whether the phenotype-centric class framework, trained primarily on *Aureobasidium* spp. and *Trichoderma reesei*, can capture morphotype profiles in fungi not represented during training. To explore this, we applied the Zulu_s3 model and the multi-group analysis pipeline to brightfield and DIC images of four additional groups: *Candida albicans* (DIC images from the Candescence dataset), *Komagataella phaffii* GS115, *K. phaffii* strain set_A, and *Aspergillus niger* conidia. None of these images contributed to any training or validation fold and was thus completely unseen by the model.

**Fig. 3:**
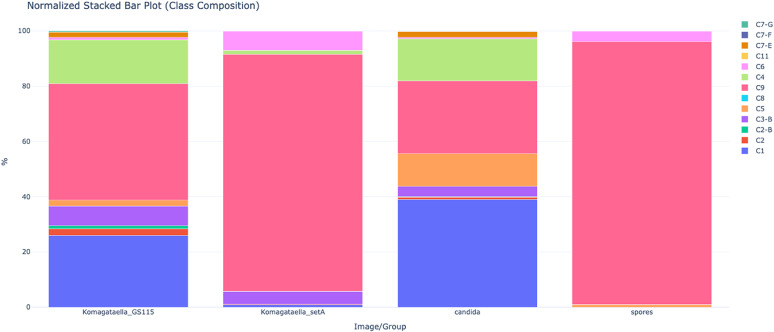
Normalized stacked bar plot representing morphological composition across four groups: *Komagataella*_GS115, *Komagataella* strain set_A, *Candida*, and spores. Each bar represents relative proportions of thirteen morphotypes, revealing population structure between groups

Across all groups, the normalised stacked bar plots (Fig. 3) show that one or two morphotypes dominate each species, but with distinct compositional profiles that reflect known biology. *A. niger* conidia were assigned almost exclusively to class C9 (spheroidal cells), consistent with the near-spherical geometry of spores. Both *K. phaffii* groups were similarly enriched for C9, with minor contributions from C3-B (cells with granular cytoplasm). In the GS115 group, a small but consistent C1 fraction (vacuolated single cells) was also present; manual inspection of a representative image confirmed that cells with faint centres and darker boundaries were classified as C1, while spherical cells were correctly assigned to C9. Septate filaments (C7-E) were detected in the GS115 group, consistent with the pseudohyphal growth this strain is known to undergo under certain cultivation conditions [35]. *K. phaffii* strain set_A was likewise enriched for C9, with minor contributions from C3-B and low counts of C6 (cell clumps).

**Fig. 4:**
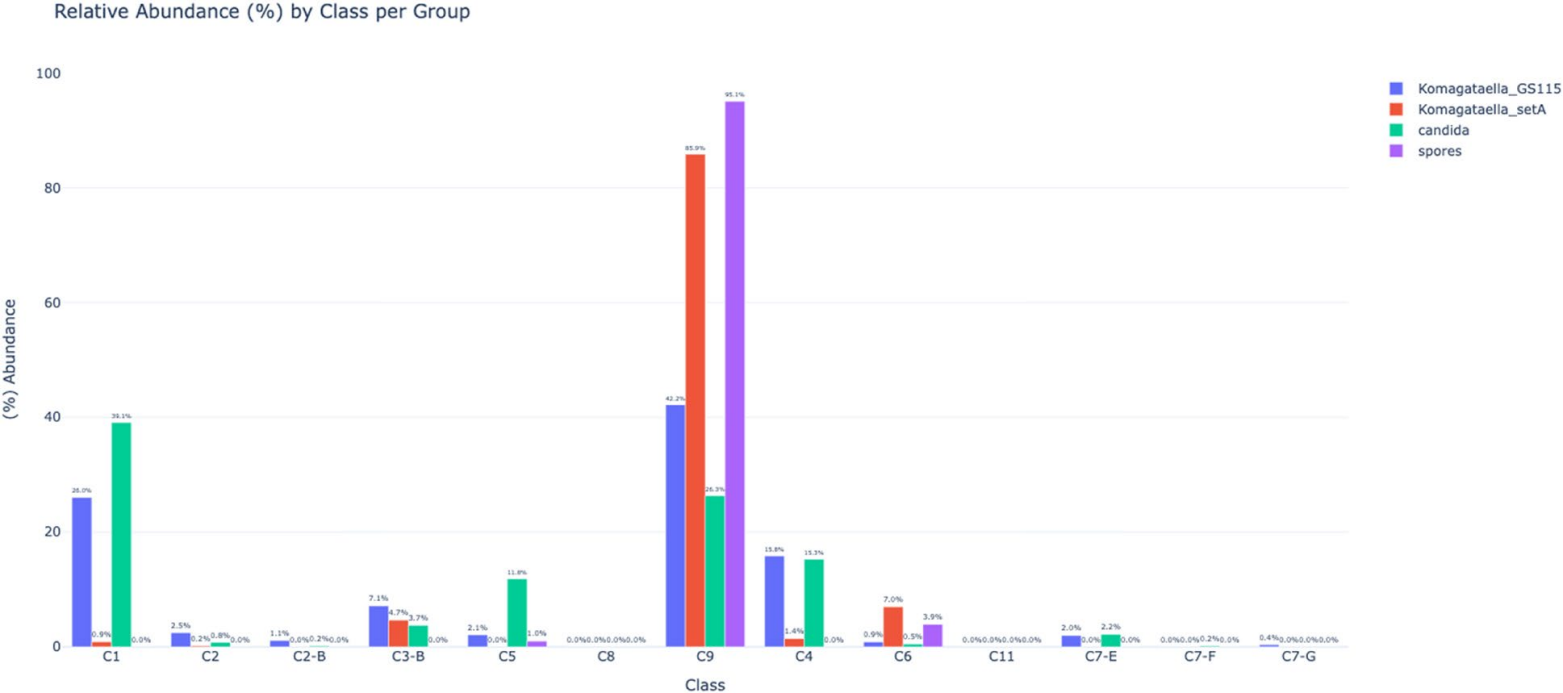
Relative abundance (%) of each morphological class per group, providing an overview of morphological composition across groups

In contrast, the *C. albicans* group exhibited a markedly more heterogeneous profile, with meaningful contributions from C9, C1, C3-B, C4 (budding events), and the hyphal classes C7-E and C7-F. This broader morphotype distribution is consistent with the well-documented polymorphic capacity of *C. albicans*, which readily transitions between yeast, pseudohyphal, and true hyphal forms depending on environmental conditions [1, 2]. These group-level differences are captured clearly in the relative (Fig. 4) and mean relative abundance plots (Supplementary Fig. 7): C9 dominates *K. phaffii* strain set_A and *A. niger*, whereas *C. albicans* and *K. phaffii* GS115 show comparatively higher proportions of C1 and C3-B, and *C. albicans* uniquely contributes filamentous and budding morphotype classes.

From a biological standpoint, these analyses suggest that TU_MyCo-Vision can be useful to extract coherent, group-level morphotype signatures from unseen genera. However, given that the model was trained primarily on *Aureobasidium.spp* & *T.reesei* images, and that class-wise performance is weaker for rare and filamentous classes, these cross-species profiles should be regarded as exploratory rather than as exact quantitative assessment or taxonomically diagnostic readouts. It is important to note that the patterns reported here for TU_MyCo Vision have not been systematically validated against existing image analysis tools or reference modalities (e.g. differences in contrast, magnification, and acquisition settings), direct one-to-one comparison of class assignments is inherently limited. Accordingly, our analyses are intended primarily to demonstrate the broader applicability and exploratory potential of TU_MyCo Vision for fungal morphotype profiling, rather than to provide an absolute performance ranking or fully harmonised correspondence with other tools. Refer to Supplementary Folder S12 (data_gui_test) for raw data, plots and predictions.

#### Validation of the data analysis suite against manually annotated labels

To assess how closely TU_MyCo-Vision-derived morphotype profiles reflect manually curated counts, the multi-group analysis pipeline was applied in parallel to model predictions (“aureo”) and manual annotations (“manual”) for the same 90 images from the independent global test set. Any differences between the two groups reflect the Zulu_s3 model’s detection performance, not errors introduced by the analysis pipeline.

**Fig. 5:**
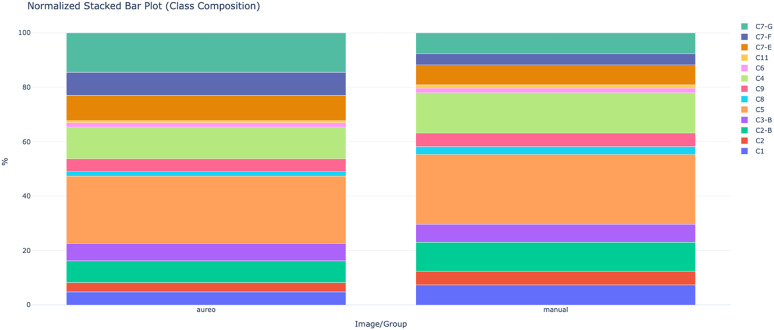
Validation of TU_MyCoVision predictions against ground-truth annotations on 90 test-set images. Model predictions (“aureo”, blue) and manually curated ground-truth annotations (“manual”, red) were compared using the multi-group analysis pipeline. Normalised stacked bar plot showing overall morphotype composition; the dominant classes: yeast-like cells (C5), budding events (C4), and filamentous morphologies (C7-E/F/G) are consistently represented in both groups

**Fig. 6:**
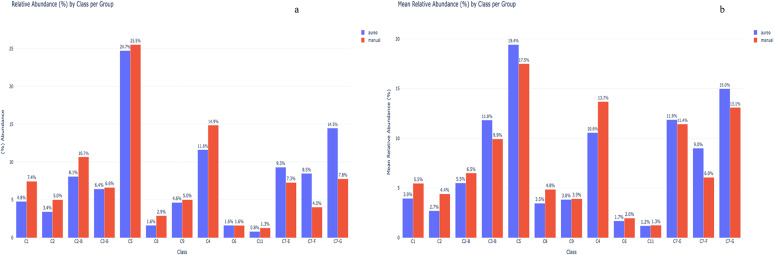
**a** Relative abundance (%) per class, highlighting the largest deviations: budding events (C4: 11.6% vs. 14.9%), vacuolated cells (C1: 4.8% vs. 7.4%; C2-B: 8.1% vs. 10.7%), and smooth filaments (C7-G: 14.5% vs. 7.8%). **b** Mean relative abundance (%), where per-image averaging reduces field-level heterogeneity and narrows deviations for most classes. Filamentous class counts should be treated as semi-quantitative, as a single hypha may be enclosed by multiple bounding boxes

The normalised stacked bar plots (Fig. 5) show that the overall compositional structure is well preserved: both groups are dominated by yeast-like cells (C5), budding cells (C4) and filamentous morphologies (C7-E, C7- & C7-G), confirming that the model captures the principal morphological character of the test population. The relative abundance plot (Fig. 6a) reveals the largest class-level discrepancies. Budding events (C4) are the most underestimated class (model: 11.6%; ground truth: 14.9%), consistent with the limited recall for this morphotype reported in cross-validation. Vacuolated single cells (C1: 4.8% vs. 7.4%; C2-B: 8.1% vs. 10.7%) and smooth filaments (C7-G: 14.5% vs. 7.8%) show the next largest deviations. By contrast, yeast-like cells (C5: 24.7% vs. 25.5%) and cells with granular cytoplasm (C3-B: 6.4% vs. 6.6%) are recovered with high fidelity.

The mean relative abundance plot (Fig. 6b) was designed to reduce the influence of image-to-image variation and provide a more stable population-level estimate. For several classes, notably irregular cells (C11) and filamentous classes, the gap between predicted and ground-truth values narrows compared to the relative abundance plot, demonstrating the utility of this metric for smoothing field-level heterogeneity. However, the improvement is not uniform: deviations for vacuolated cells (C2-B) and yeast-like cells (C5) remain or widen slightly, indicating that mean relative abundance does not fully compensate for systematic recall deficiencies.

A specific caution applies to filamentous classes (C7-E, C7-F, C7-G): because a single continuous hypha may be enclosed by multiple bounding boxes, raw detection counts can overestimate filament abundance. Users are therefore advised to treat filamentous morphotype values as qualitative indicators of presence and abundance rather than as precise quantitative estimates. This issue could also be mitigated by processing more images through the model.

Taken together, these comparisons confirm the potential of TU_MyCo-Vision to capture the dominant morphotype composition of a population from brightfield images. Class-specific estimates, particularly for budding events, vacuolated single cells, and filamentous morphotypes, should be interpreted with caution and awareness of the model’s performance limitations, and conclusions should be drawn from trends across multiple images rather than from individual field counts.

## Conclusion

TU_MyCo-Vision addresses a practical and recurring challenge in fungal biology: the consistent, high-throughput quantification of morphotype diversity from brightfield microscopy images. Manual assessment of morphotype distributions across large image sets is labour-intensive, subjective, and prone to inter-observer variability, limitations that become particularly acute in organisms like *Aureobasidium pullulans*, where pronounced morphological plasticity is observed across strains, media, and cultivation conditions.

To address this, we defined thirteen biologically grounded morphotype classes from brightfield microscopy observations and trained a YOLOv11m-based object detector across three iteratively refined datasets. The final model, Zulu_s3, achieves reliable detection for the majority of single-cell morphotypes, with performance characteristics suitable for population-level morphotype quantification. Detection of filamentous and morphologically ambiguous classes remains more challenging, and outputs for these classes should be treated as qualitative indicators of presence rather than precise counts.

A cross-platform GUI integrates detection with a quantitative analysis pipeline, enabling morphotype quantification, group comparisons, and interactive visualisation without programming expertise. An exploratory application to *C. albicans*, *K. phaffii*, and *A. niger*, none of which contributed to training, suggests that the phenotype-centric class framework generalises beyond *Aureobasidium*; cross-species results should be regarded as exploratory at this stage.

TU_MyCo-Vision is intended as a practical starting point for automated morphotype profiling in fungi, lowering the barrier to quantitative microscopy for researchers without image analysis expertise. Please refer to the supplementary file “worked example” for a detailed walk-through on how to use TU_MyCo-Vision.

## Supplementary Information

Below is the link to the electronic supplementary material.

Supplementary Material 1.

Supplementary Material 2.

Supplementary Material 3.

Supplementary Material 4.

Supplementary Material 5.

Supplementary Material 6.

Supplementary Material 7.

Supplementary Material 8.

Supplementary Material 9.

## Data Availability

All codes used during TU_MyCo-Vision development, standalone executables for Windows OS and MacOS, along with the model weight and supplementary files, are available on our GitHub repository at https://github.com/cderntl/TU_MycoVision. The X-ray, Yankee, Zulu, global test dataset, and test set for GUI testing are available on our Zenodo repository at https://doi.org/10.5281/zenodo.16274901. Supplementary information folders S7-S13 are available on our Zenodo repository at https://doi.org/10.5281/zenodo.16318684.

## Abbreviations

AFP: *A. flavus and A. parasiticus* medium
ARCH: Archimycetes medium
BHI: Brain Heart Infusion medium
CH: Chlamydospore-like cells
DIC: Differential Interference Contrast
FCOS: Fully Convolutional One Stage
GUI: Graphical user interface
HY: Hyphal forms
MA: Mandels–Andreotti medium
MEX: Malt extract medium
MH: Muller Hinton broth
MS: Meristematic structures
PD: Potato dextrose medium
SC: Swollen cells
SSC: Septate swollen cells
SOTA: State of the Art
YL: Yeast-like cells
YLB: Yeast-like blastospores
YOLO: You Only Look Once
YPD: Yeast peptone dextrose medium
