# Supplementary material for: Fungal morphotype detection and quantification in microscopic images with TU_MyCo-vision: a user-friendly deep learning object detection tool
Source: Fungal Biol Biotechnol. 2026 Aug 11;13:14. doi: 10.1186/s40694-026-00215-1 (PMC13459361; doi:10.1186/s40694-026-00215-1)
Supplement: Supplementary file 1 [file 40694_2026_215_MOESM8_ESM.pdf]

Supplemental Figures

Fungal Morphotype Detection and Quantification in Microscopic Images with TU_MyCo-Vision, a User-Friendly Deep Learning Object Detection Tool

Kartik J. Deopujari[1], Matthias Schmal[1], Caroline Danner[1], Zainab Abdul Qayyum[1], Jordy T. Zwerus[1], Julian Kopp[1], Mihail Besleaga[1], Roghayeh Shirvani[1], Astrid R. Mach-Aigner[1], Robert L. Mach[1], Christian Zimmermann[1*]

[1] Institute of Chemical, Environmental and Bioscience Engineering, TU Wien, Gumpendorfer Strasse 1a, 1060 Wien, Austria

* corresponding author: christian.zimmermann@tuwien.ac.at

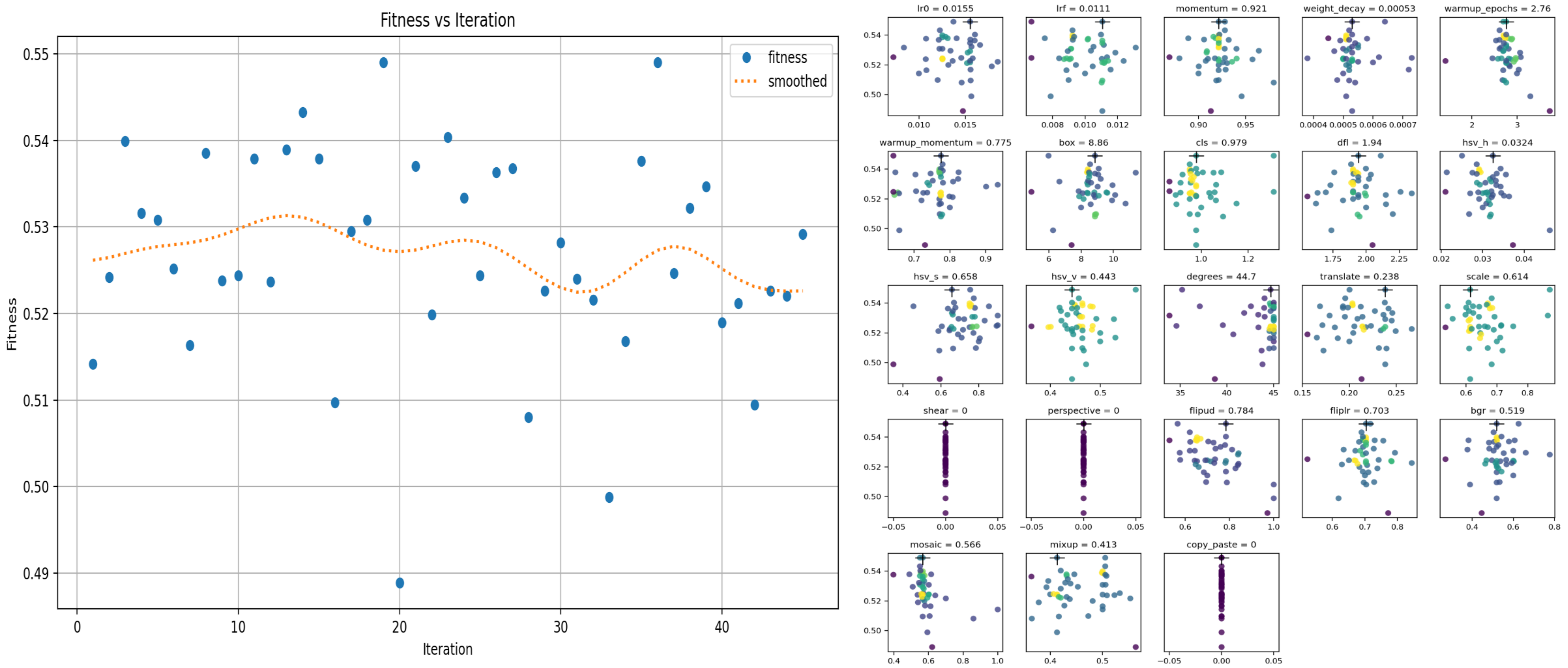

Figure 1. (Left Panel) Fitness vs. iteration plot. Fitness scores across 45 iterations of hyperparameter tuning. The dotted line indicates a smoothed trend. Scatter plots of tuned hyperparameters vs. fitness. (Right Panel) Each subplot shows the relationship between the hyperparameter and resulting fitness scores during the hyperparameter evolution. Annotated values indicate the best-performing parameters. Parameters set to ‘0’ were not tuned during the hyperparameter evolution.

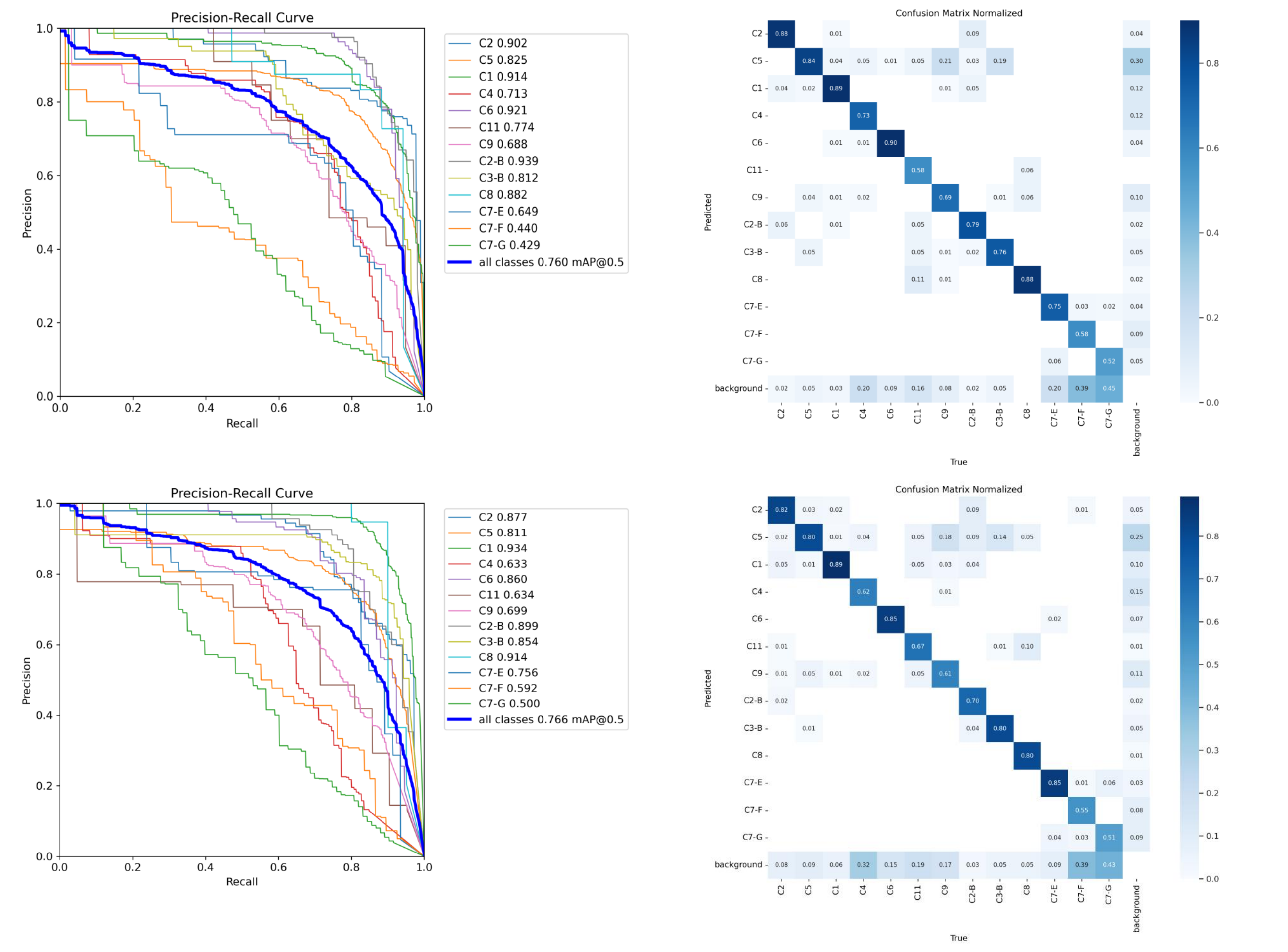

Figure 2. Detection performance of X-ray and Yankee model families.

Precision-Recall curves (left) and normalised confusion matrices (right) for the best-performing models from the X-ray (top; xray_split4, mAP@50 = 0.760) and Yankee (bottom; yankee_split5, mAP@50 = 0.766) dataset families. Diagonal values in the confusion matrices represent correct classification rates; the bold blue PR curve indicates the mean across all classes. Transition from X-ray to Yankee yielded the most notable improvement for septate filaments (C7-E; mAP@50: 0.649 → 0.756), while vacuolated (C7-F) and smooth filaments (C7-G) remained the most challenging classes in both families

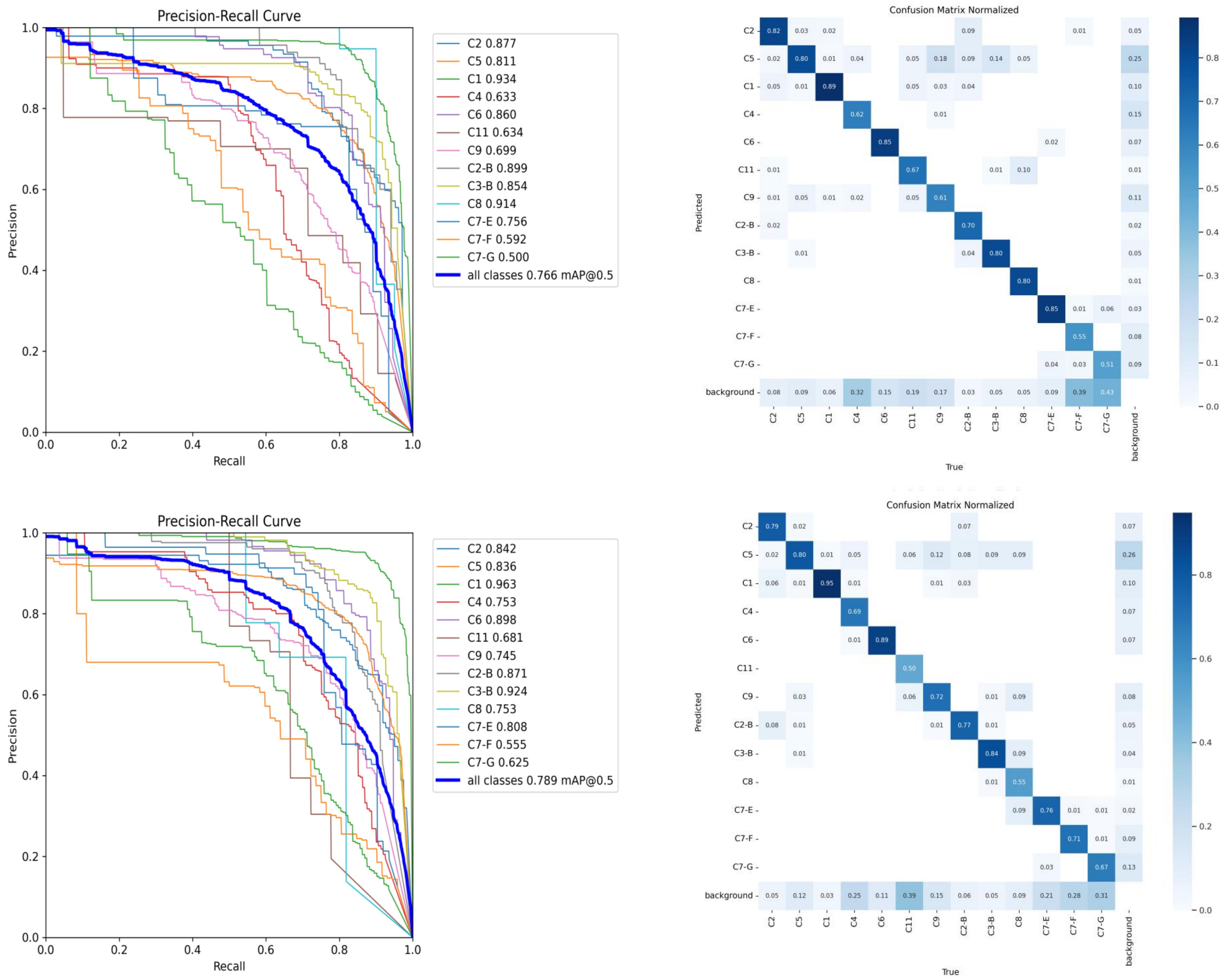

Figure 3. Detection performance of Yankee and Zulu model families.

Precision-Recall curves (left) and normalised confusion matrices (right) for the best-performing models from the Yankee (top; yankee_split5, mAP@50 = 0.766) and Zulu (bottom; zulu_split1, mAP@50 = 0.789) dataset families. Transition from Yankee to Zulu yielded broad improvements across all morphotype classes, most notably for smooth filaments (C7-G; mAP@50: 0.500 → 0.625), vacuolated single cells (C1; 0.934 → 0.963), and granular cytoplasm cells (C3-B; 0.854 → 0.924), reflecting the targeted enrichment of underrepresented classes in the Zulu dataset.

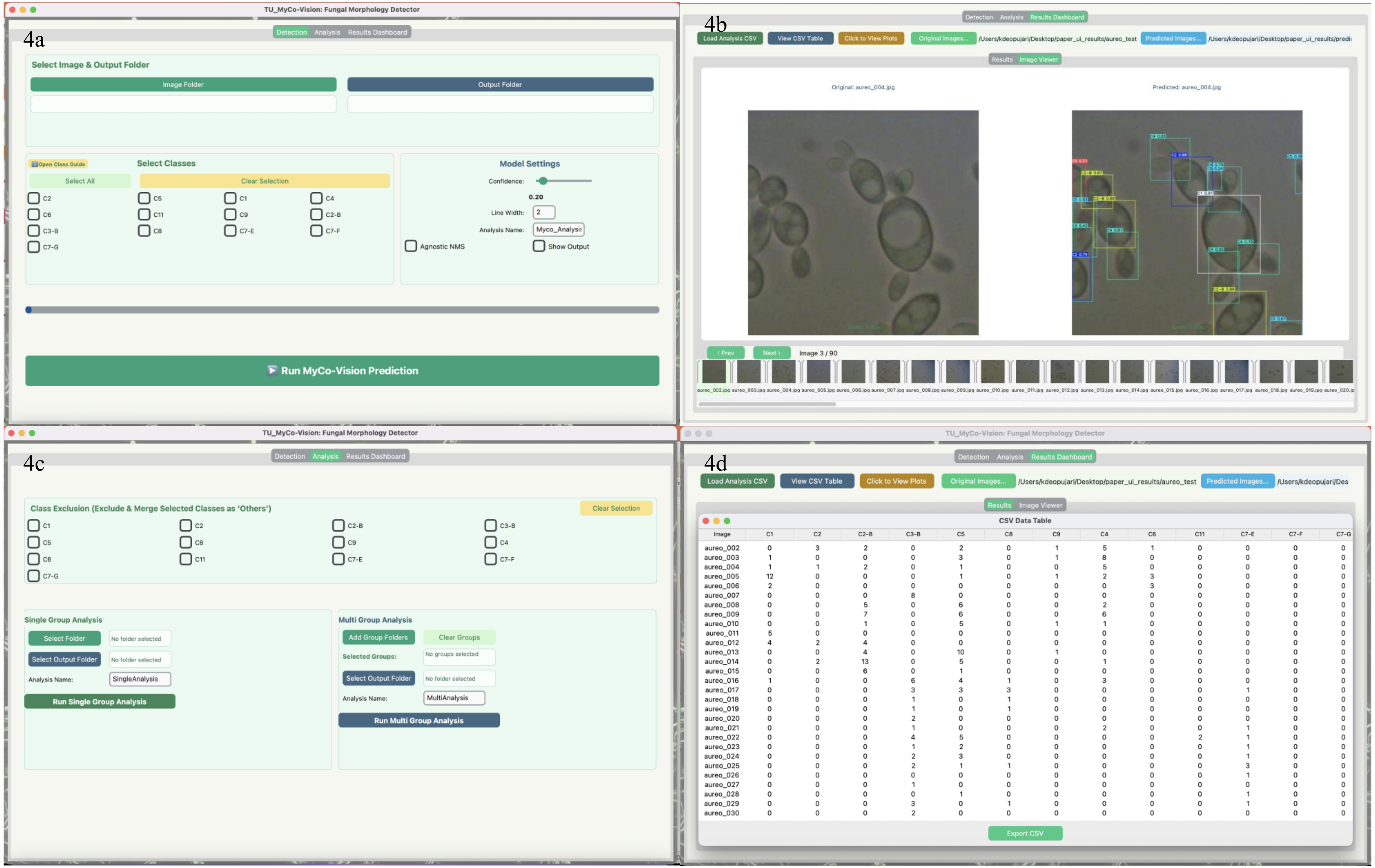

Figure 4. (a) Detection panel, enabling users to select input/output folders, select morphological classes, adjust model parameters, and execute prediction. (b) Result Dashboard predictions viewer displaying paired original and annotated images side-by-side, with navigation panel. (c) Analysis panel, which includes the class exclusion feature and options for single-group and multi-group analysis, along with the option to select input/output folders and analysis name. (d) Results Dashboard panel CSV data table view.

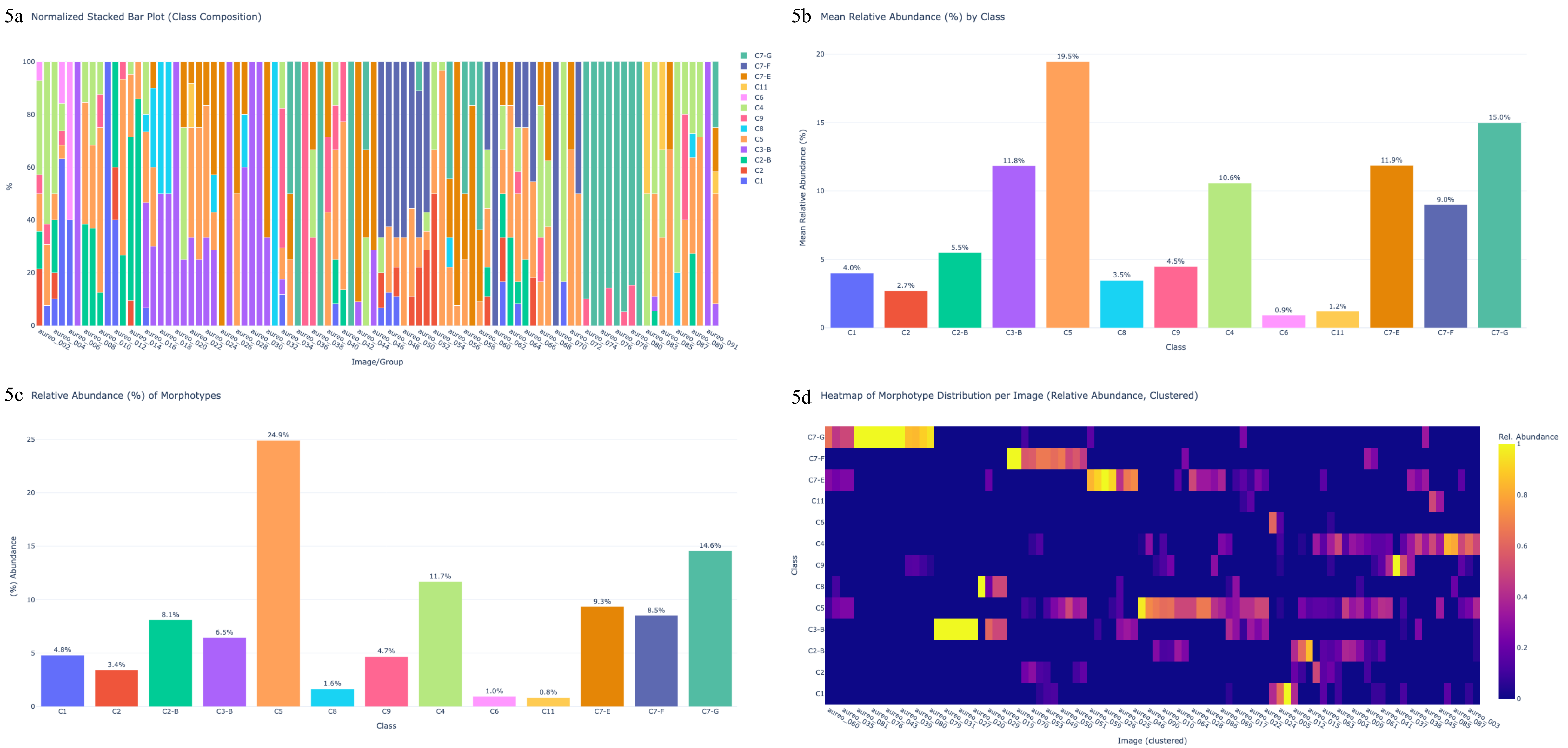

Figure 5. (a) Normalized stacked bar plot representing morphological composition across 90 test set images. (b) Mean relative abundance (%) of each morphological class across all images. (c) Relative abundance (%) of each morphological class across the dataset. (d) Relative abundance based hierarchically clustered heatmap.

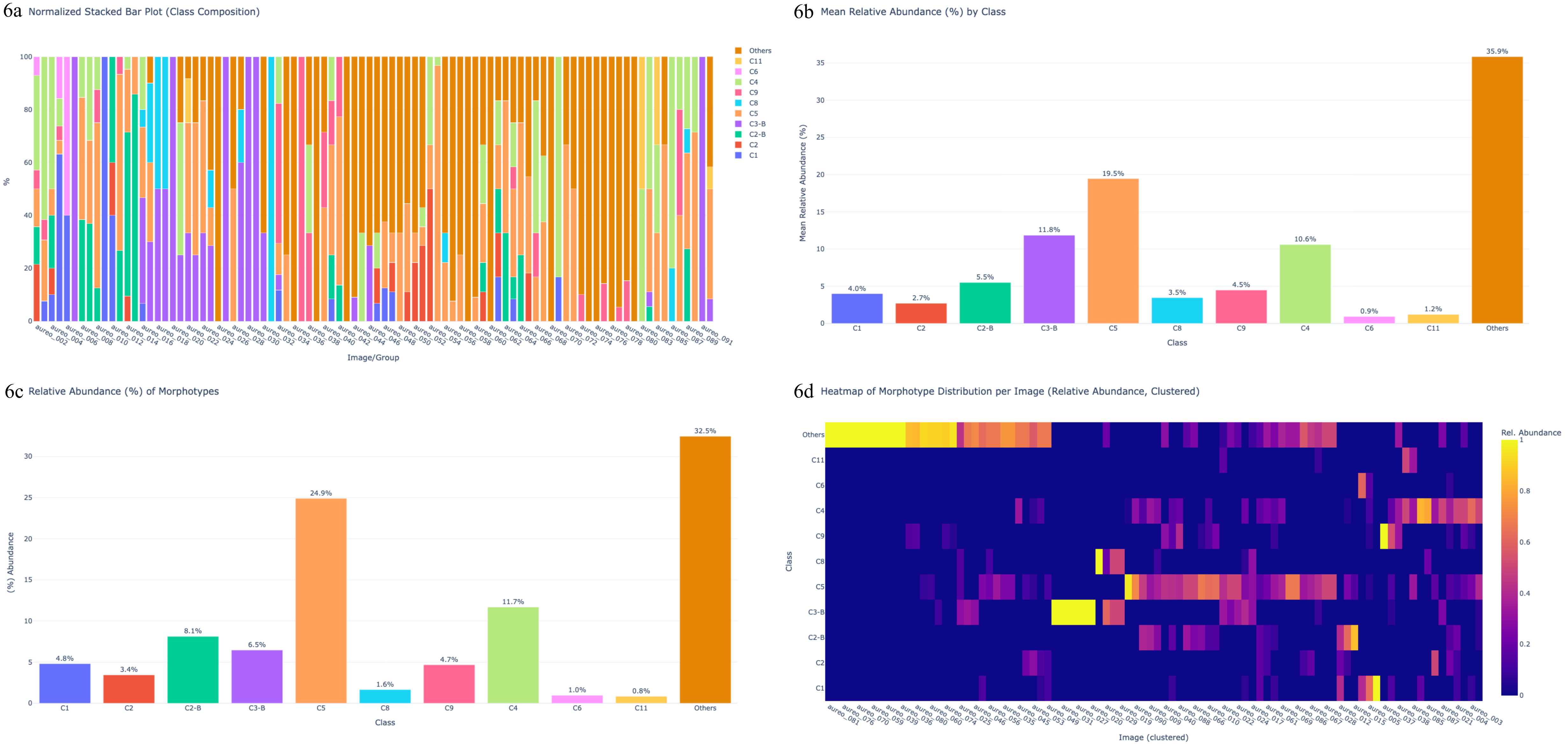

Figure 6. (a) Normalized stacked bar plot of morphological composition in 90 test set images after enabling class exclusion, where C7-E, C7-F, and C7-G (filamentous morphologies) are grouped into the “Others” class. (b) Mean relative abundance (%) of morphological classes after applying class exclusion. (c): Relative abundance (%) of classes post-class exclusion. (d) Relative abundance based hierarchically clustered heatmap post-class exclusion.

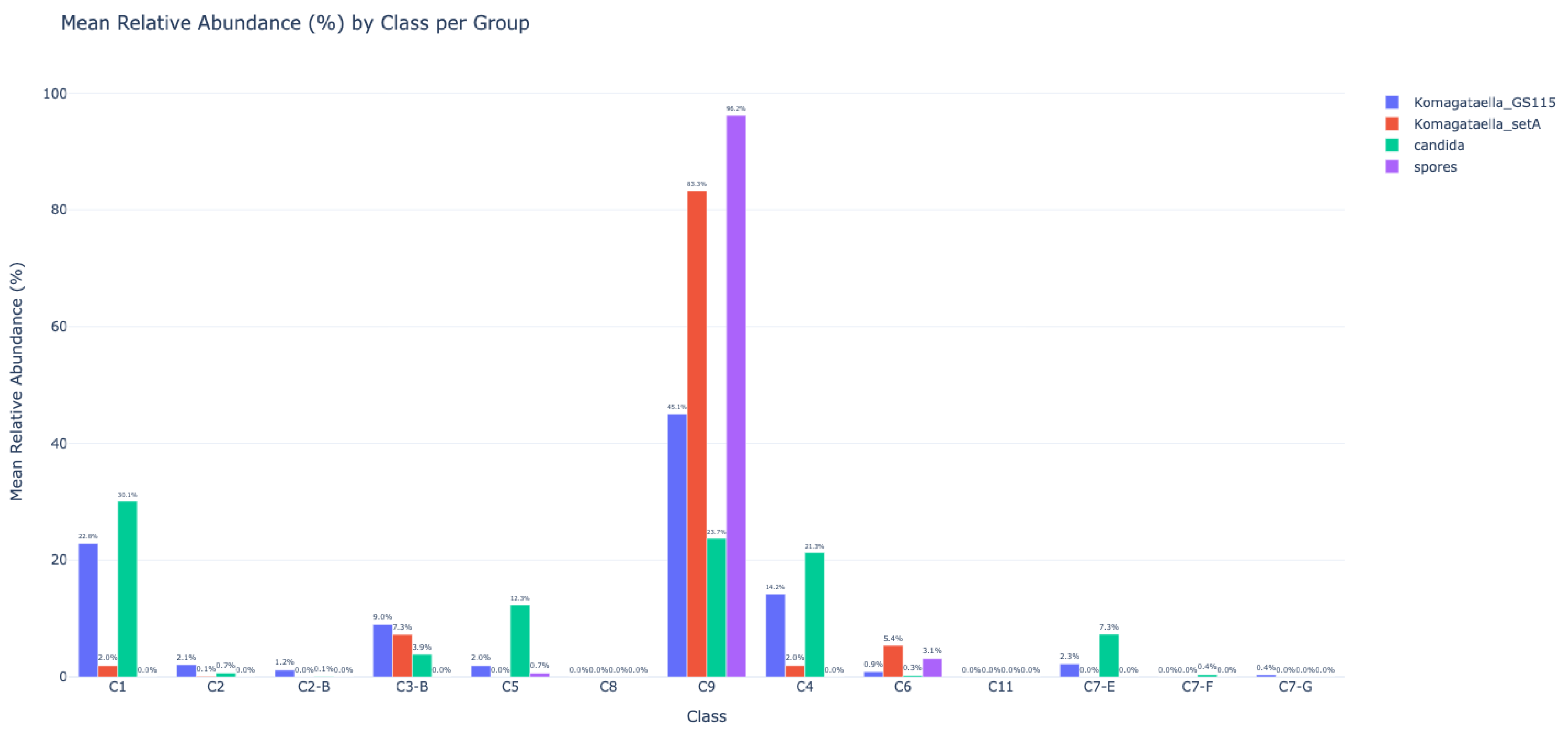

Figure 7. Mean relative abundance (%) of each morphological class per group, providing an overview of morphological composition across groups.
