# Supplementary material for: Fungal morphotype detection and quantification in microscopic images with TU_MyCo-vision: a user-friendly deep learning object detection tool
Source: Fungal Biol Biotechnol. 2026 Aug 11;13:14. doi: 10.1186/s40694-026-00215-1 (PMC13459361; doi:10.1186/s40694-026-00215-1)
Supplement: Supplementary file 2 [file 40694_2026_215_MOESM9_ESM.pdf]

Worked example: Morphological variation in *A. pullulans* & Technical Demonstration of TU_MyCo-Vision

After cultivation of *A.pullulans* strains as described in Methods (Section 1) we observed a diverse set of cell morphologies that were grouped into thirteen morphotype classes as defined previously. To illustrate the biological relevance of these morphotypes and the applicability of TU_MyCo-Vision to such data, we analysed a small representative subset of images from a set of experiments with *A. pullulans* EXF-150 grown in *A.flavus* and *A.parasiticus* (AFP) medium and potato dextrose (PD) medium, images were captured at 24h, 48h, and 72h post incubation. The detailed design and biological interpretation of this cultivation experiment is out of the scope of this article and are not discussed further. The images are used solely as an application example for TU_MyCo-Vision and not for model performance assessment, as parts of the dataset overlap with the training and validation images.

1.1. Temporal morphological shift in AFP medium

In AFP medium, TU_MyCo-Vision captures the time-dependent morphological change across three time points. In Fig.1 (a-c) images are ordered as 24h, 48h and 72h respectively, Fig.1(d-f) shows the corresponding TU_MyCo-Vision detection output with class-specific bounding boxes. The Relative abundance plot for *A.pullulans* EXF-150 (prefix: AFP_150) (Fig. 2a) shows that the AFP subset is dominated by C1 cells, which account for 47% of all detections. C5 (yeast like cells) form the second largest group at 25.3% followed by C4 (budding events). Other classes C3-B, C8, C11 filamentous classes (C7-E/F/G) were not detected. This indicated that the population is dominated by vacuolated single cell classes and yeast like cells (C5). The normalized stacked bar plot (Fig. 2b) for AFP cultivation reveals a temporal change in the morphological composition of the culture. At 24h point the C5 cell type dominates the culture, followed by budding events (C4). By 48h, the composition shifts and dominated by C1 (cell

with single central vacuole) cells, followed by C2 (dual vacuoles, binocular appearance). At 72h, the bar is almost entirely composed of C1 cell types, with a small proportion of other classes, indicating the predominance of vacuolated single cells at late time points. Taken together, these plots show that TU_MyCo-Vision can resolve a transition from normal yeastlike cells (C5) towards a population dominated by vacuolated cell types (C1, C2).

1.2. Stable morphotype composition in PD medium

For *A.pullulans* EXF-150 cultivated in PD medium (Prefix: PD_150),

In Fig.3 (a-c) images are ordered as 24h, 48h and 72h respectively, Fig.3(d-f) shows the corresponding TU_MyCo-Vision detection output with class-specific bounding boxes. The relative abundance (%) plot (Fig. 2c) indicates distinct composition when compared to AFP medium. C3-B, cells with granular cytoplasm is the most abundant class (41%) followed by yeast like cells (C5). One contrasting feature is the presence of filamentous morphologies (C7-E) i.e. septate hypha/pseudohypha, which accounts for 16.8% of population. However, do not recommend the absolute quantification of filamentous morphologies and use this as a qualitative assessment. The normalized stacked bar plot (Fig. 3d) indicates that morphological profile is relatively stable throughout the cultivation, with minor variation is the population of C3-B cells. These PD plots therefore indicate that, under the conditions tested, PD medium maintains a stable granular–filamentous regime with coexisting C3-B, C5, and C7-E, rather than driving the culture towards a single dominant morphotype.

1.3. Medium dependent morphotype composition

We compared the AFP and PD cultures based on the Normalized Stacked Bar Plot (Fig. 4a) and Mean Relative Abundance (%) (Fig. 4b). The most contrasting difference is the presence of C1 cell type in AFP (43.2%) and C3-B in PD (44%), along with the filamentous morphology present in PD medium as opposed to all single celled in AFP. In the images tested C1 remains

undetected in PD and C3-B remains undetected in AFP medium. The proportion of C5 cells (yeast like) is comparable between the two media, indicating it as the "always present" cell type in both the conditions.

Collectively, these values highlight that APF is characterized by vacuolated single-cell dominance (C1, C2, C2-B) and PD is characterized by granular cell (C3-B) and filamentous morphologies (C7-E).

TU_MyCo-Vision thus provides a concise, quantitative summary of medium-dependent morphotype regimes from a limited number of images.

1.4. Interpretation and limitation of this demonstration

Because all or part of the images used in this example were included in the TU_MyCo-Vision training dataset, the resulting predictions and plots do not represent an independent validation of model performance. Instead, these images and their derived summaries are provided solely as a technical demonstration of how TU_MyCo-Vision can be applied to (i) quantify temporal changes in morphotype composition within a single medium and (ii) compare different cultivation conditions to identify contrasting morphological differences or overall population trends.

In addition, the values indicated in the bar plots should not be interpreted as the exact state of the culture, because the underlying numbers and class distributions are expected to change as more images are incorporated and are also subject to the inherent prediction errors and misclassifications of the TU_MyCo-Vision model itself. Each plot is based on a limited number of microscopic images, and the observed differences between time points or media may partly reflect variation in local cell density and field selection rather than true culture-wide shifts. For robust biological conclusions, it is therefore recommended to analyze as many independent images as practically feasible, ideally sampled systematically across the culture, so that

TU_MyCo-Vision outputs represent the global morphotype distribution rather than field-to-field variability.

Image Captions

Figure 1. (a-c) Microscopic images of *A. pullulans* EXF-150 cultivated in AFP medium. Left to right 24h to 72h post incubation (Total magnification = 200x). (d-e) TU_MyCo-Vision detection results of images (a-b). Objects are enclosed with bounding boxes of corresponding classes (Cyan – C5, White – C1, Blue – C2, Dark Blue – C6, Yellow – C2-B).

Figure 2. (a-b) Relative % abundance & Normalized stacked bar plot representing the temporal dynamics of *A. pullulans* EXF-150 cultivated in AFP medium. In Normalized stacked plot (left to right) indicated transition of 24-72h incubation. (c-d) Relative % abundance & Normalized stacked bar plot representing the morphological profile of *A. pullulans* EXF-150 cultivated in PD medium. In Normalized stacked plot (left to right) indicated 24-72h incubation.

Figure 3. (a-c) Microscopic images of *A. pullulans* EXF-150 cultivated in PD medium. Left to right 24h to 72h post incubation (Total magnification = 200x). (d-e) TU_MyCo-Vision detection results of images (a-b). Objects are enclosed with bounding boxes of corresponding classes (Cyan – C7-E & C5, Green – C3-B, Pink – C11, Blue – C2).

Figure 4. (a) Normalized stacked bar plot representing the overall differences in morphological profile of *A.pullulans* EXF-150 cultivated in AFP and PD medium. (b) Mean Realtive % Abundance plots for representing class wise distribution in each individual group (AFP vs PD).

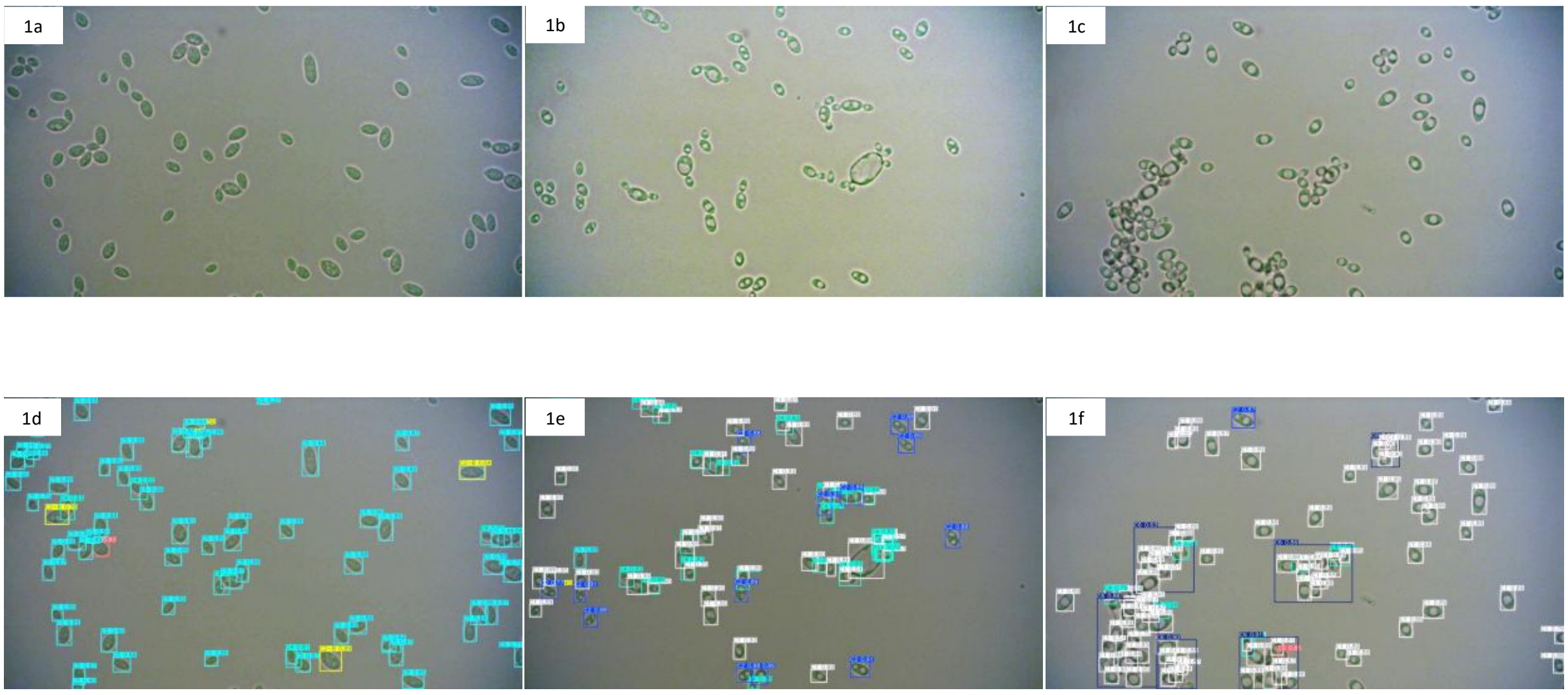
1a
1b
1c
1d
1e
1f

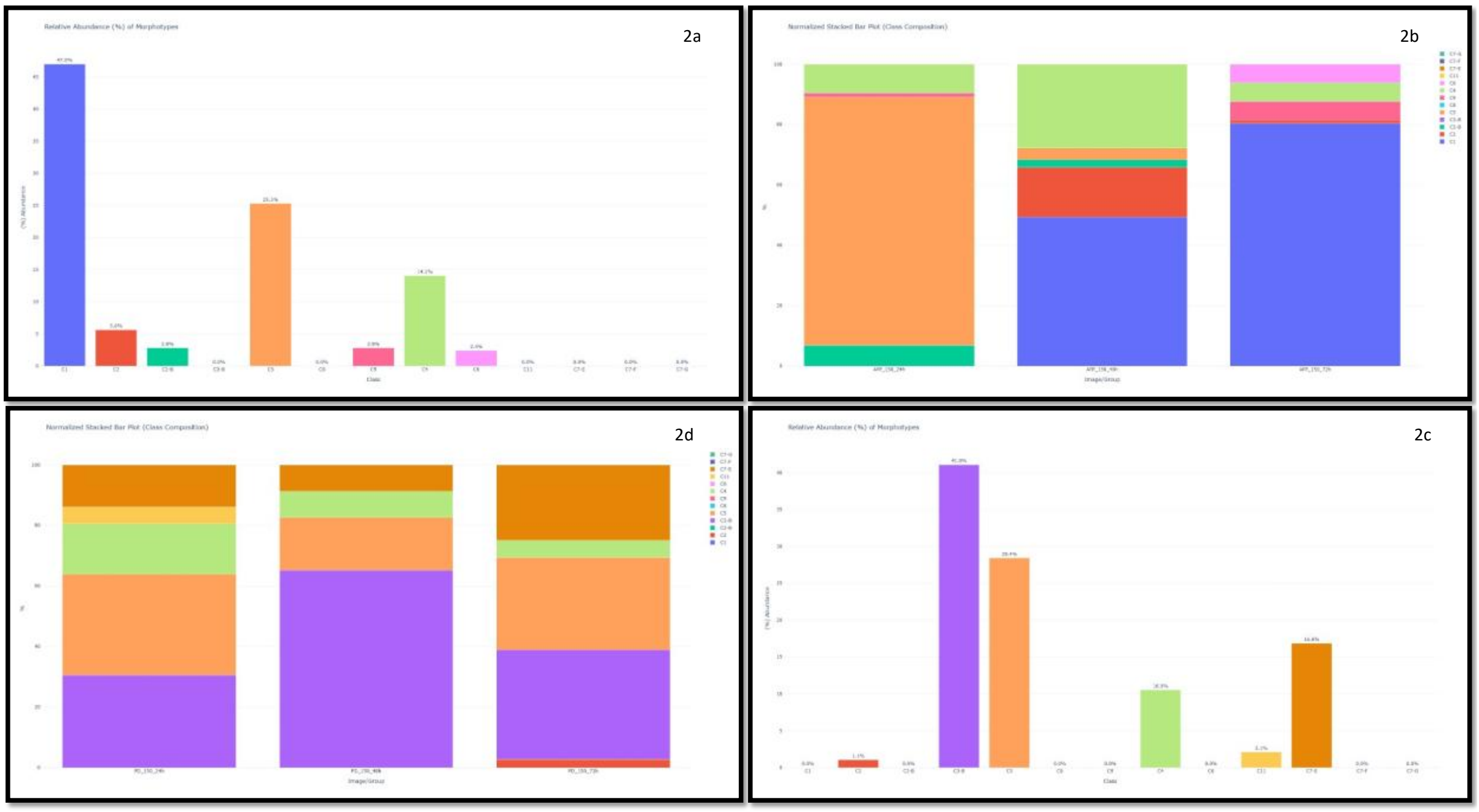
2a
Relative Abundance (%) of Morphotypes
2b
Normalized Stacked Bar Plot (Class Composition)
2d
Normalized Stacked Bar Plot (Class Composition)
2c
Relative Abundance (%) of Morphotypes

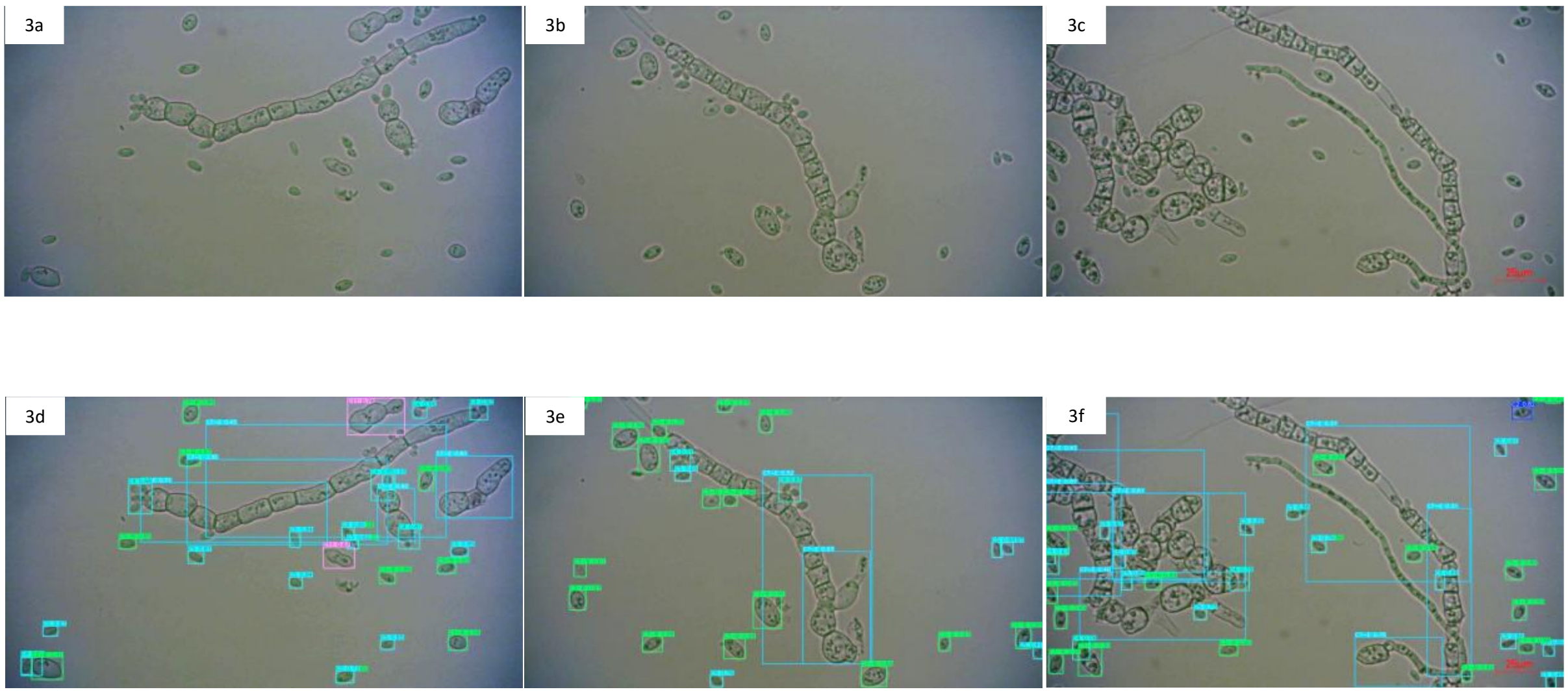
3a
3b
3c
3d
3e
3f

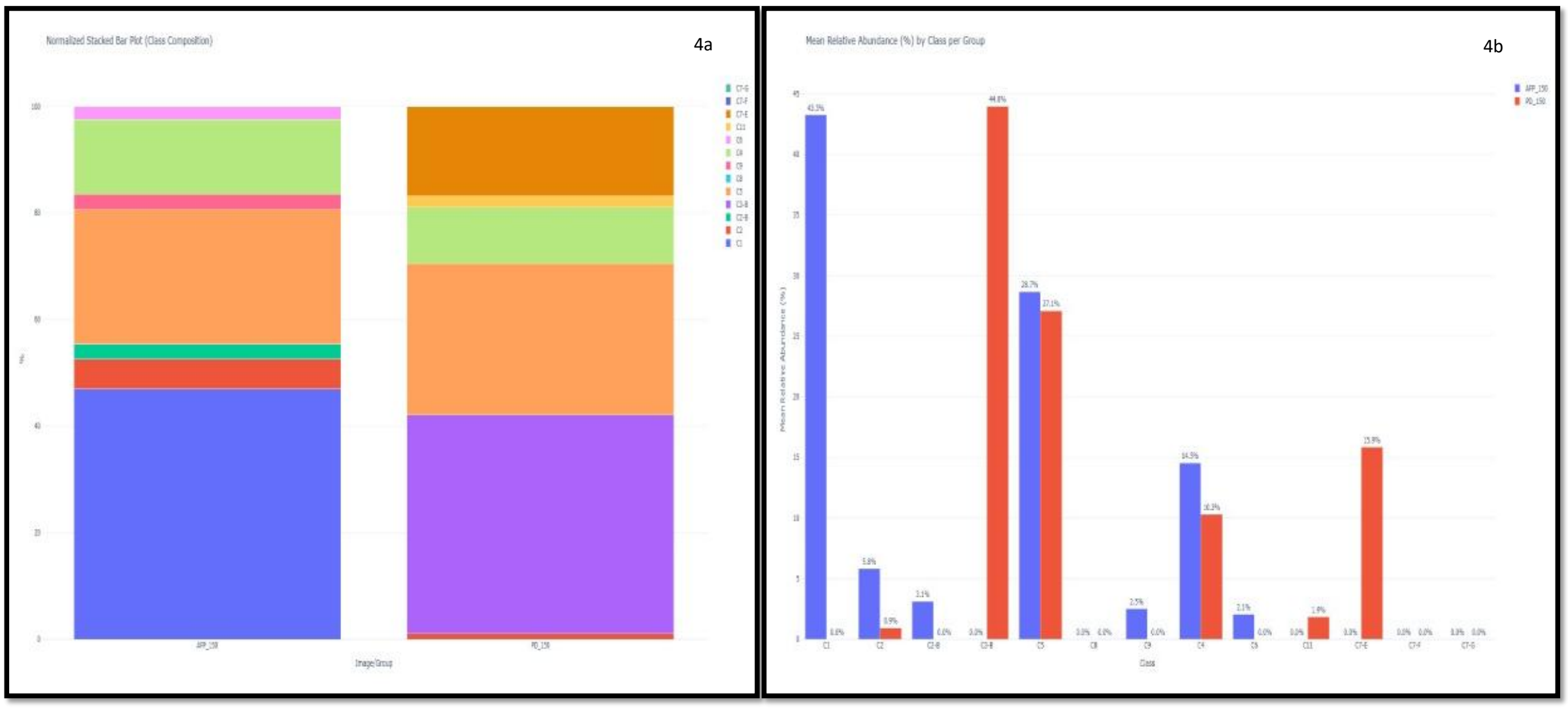
Normalized Stacked Bar Plot (Class Composition)
4a
Mean Relative Abundance (%) by Class per Group
4b
